# Integration of transcriptomics and metabolomics reveals the responses of the maternal circulation and maternal-fetal interface to LPS-induced preterm birth in mice

**DOI:** 10.3389/fimmu.2023.1213902

**Published:** 2023-08-15

**Authors:** Xianling Cao, Xuanyou Zhou, Songchang Chen, Chenming Xu

**Affiliations:** ^1^ Obstetrics and Gynecology Hospital, Institute of Reproduction and Development, Fudan University, Shanghai, China; ^2^ Research Units of Embryo Original Diseases, Chinese Academy of Medical Sciences, Shanghai, China; ^3^ Key Laboratory of Reproductive Genetics (Ministry of Education), Department of Reproductive Endocrinology, Women’s Hospital, Zhejiang University School of Medicine, Hangzhou, China

**Keywords:** maternal-fetal interface, transcriptomics, metabolomics, preterm birth, mice

## Abstract

**Background:**

Term birth (TB) and preterm birth (PTB) are characterized by uterine contractions, rupture of the chorioamniotic membrane, decidual activation, and other physiological and pathological changes. In this study, we hypothesize that inflammation can cause changes in mRNA expression and metabolic stability in the placenta, decidua, chorioamniotic membrane, uterus and peripheral blood, ultimately leading to PTB.

**Methods:**

To comprehensively assess the effects of inflammation on mRNA expression and metabolite production in different tissues of pregnancy, we used a mouse PTB model by intraperitoneally injecting lipopolysaccharide (LPS) and integrated transcriptomics and metabolomics studies.

**Results:**

Our analysis identified 152 common differentially expressed genes (DEGs) and 8 common differentially expressed metabolites (DEMs) in the placenta, decidua, chorioamniotic membrane, uterus, and peripheral blood, or placenta and uterus after LPS injection, respectively. Our bioinformatics analysis revealed significant enrichment of the NOD-like receptor signaling pathway (mmu04621), TNF signaling pathway (mmu04668), IL-17 signaling pathway (mmu04657), and NF-kappa B signaling pathway in the transcriptomics of different tissues, and Hormone synthesis, Lysosome, NOD-like receptor signaling pathway, and Protein digest and absorption pathway in metabolomics. Moreover, we found that several upstream regulators and master regulators, including STAT1, STAT3, and NFKB1, were altered after exposure to inflammation in the different tissues. Interaction network analysis of transcriptomics and metabolomics DEGs and DEMs also revealed functional changes in mice intraperitoneally injected with LPS.

**Conclusions:**

Overall, our study identified significant and biologically relevant alterations in the placenta, decidua, chorioamniotic membrane, uterus, peripheral blood transcriptome and the placenta and uterus metabolome in mice exposed to LPS. Thus, a comprehensive analysis of different pregnancy tissues in mice intraperitoneally injected with LPS by combining transcriptomics and metabolomics may help to systematically understand the local and systemic changes associated with PTB caused by inflammation.

## Introduction

Preterm birth (PTB) is a condition where delivery occurs before 37 weeks of pregnancy. According to the World Health Organization, the global incidence of PTB is approximately 10%, and it is a leading cause of perinatal morbidity and mortality, with an estimated 15 million premature infants born worldwide each year (1–5). Although the withdrawal of progesterone, secretion of oxytocin, activation of decidual inflammation, and fetal immune response are believed to be involved in the onset of term labor (TB), the pathogenesis and etiology of PTB remain unclear (3).

PTB is widely believed to be an inflammatory process, and epidemiological, clinical, and animal studies have established a correlation between inflammation and PTB (6–8). The onset of labor in both rodents and humans involves positive feedback from the inflammatory cascade, driven by increased levels of pro-inflammatory cytokines, chemokines, and prostaglandins (PGs) at the maternal-fetal interface (9–11). The closest position between the mother and the fetus is the maternal-fetal interface, which comprises the placenta, decidua, uterus and chorioamniotic membrane. Toll-like receptors (TLRs) are critical upstream gatekeepers of inflammation, which are highly expressed at the maternal-fetal interface. Studies have shown that premature activation of TLR4 signaling at the maternal-fetal interface can lead to PTB (12, 13). In addition, damage-associated molecular patterns (DAMPs) and pathogen-associated molecular patterns (PAMPs), the endogenous and microbe-released TLR ligands, respectively (13), activate TLR4 to trigger the release of downstream pro-inflammatory cytokines and chemokines such as IL-1β, IL-6, IL-8, CCL5, and TNF-α, leading to leukocyte infiltration at the maternal-fetal interface and cervix, as well as promoting the rupture of membranes, cervical ripening and uterine contraction by activating the release of matrix metalloproteinases (MMPs) and PGs (14).

The maintenance of human pregnancy and initiation of delivery has been closely linked to the dynamic balance of the immune microenvironment at the maternal-fetal interface (15, 16). During delivery, immune cells present at the maternal-fetal interface create an inflammatory environment that facilitates the expulsion of the fetus from the mother. However, disruption of the immune balance at the maternal-fetal interface can result in premature activation of the common pathway of delivery, leading to PTB (15–17). Besides, clinical and animal studies have shown a significant positive correlation between infection, inflammation, and PTB (18). Microorganisms associated with PTB are typically derived from the reproductive or urogenital tract or blood and secrete endotoxins such as lipopolysaccharide (LPS) at the maternal-fetal interface, causing intense inflammation and ultimately resulting in premature uterine contraction, rupture of fetal membranes, and cervical ripening (14). Therefore, maternal-fetal interface immune imbalance leading to defects in inflammatory factor release and metabolic stability may play a crucial role in the process of PTB. However, the pathogenesis of PTB and the role of inflammation in causing labor have yet to be fully elucidated, and current drug treatments, including antibiotics, for PTB are not entirely satisfactory (8).

Although the use of high-throughput omics techniques has generated a vast amount of omics data, it is challenging to fully understand complex biological processes and the regulation of biological networks using just one type of omics data. By integrating transcriptomic and metabolomic analyses, we can systematically and thoroughly examine the functions and regulatory mechanisms of biomolecules, ultimately identifying key metabolic pathways, genes, and metabolites for further in-depth research and application. Thus, this present study was designed to identify new pathways in the maternal-fetal interface that undergo significant changes in an acute inflammatory environment and provide new insights into the potential pathological mechanisms of PTB by exploring the role of the maternal-fetal interface in the initiation of labor mediated by inflammatory responses through the evaluation of the transcriptomes of maternal peripheral blood, placenta, uterus, decidua, and chorioamniotic membrane, as well as the placental and uterine metabolomes, in an LPS-induced PTB mouse model.

## Materials and methods

### Mice

C57BL/6 inbred, timed pregnant mice were purchased from the *GemPharmatech* Co., Ltd (Jiangsu, China). Briefly, the mice were housed under a 12-hour light: 12-hour dark circadian cycle and female mice aged 8-12 weeks were mated with fertile male mice. We examined the females daily between 8:00 a.m. and 9:00 a.m. and considered the appearance of a vaginal plug as 0.5 days post coitum (dpc). After separation from the males, pregnancy was confirmed by a weight gain of ≥ 2g by 12.5dpc. All animal procedures were performed in accordance with the *Principles of Laboratory Animal Care of the Department of Laboratory Animal Science* at *Fudan University* and approved by the *Ethics Committee of Fudan University* (Protocol Number: 20211203S).

### LPS model of PTB

On 16.5 dpc, mice were intraperitoneally (i.p.) injected with either 100μg/animal of lipopolysaccharide (LPS) (*Escherichia coli* O55:B5, Sigma-Aldrich, St. Louis, MO, L2880) in 200μL of sterile phosphate-buffered saline (PBS) or 200ul/animal of PBS alone as a control (n=4 per group). In this study, PTB was defined as delivery before 18.5dpc. Also, we found that a dose of 100μg of LPS was the lowest dose that reproducibly induced PTB without significant morbidity or mortality (19–22). The duration of labor was measured by recording the number of hours that passed from the time of intraperitoneal (i.p.) injection of LPS/PBS to the delivery of the first pup. **Figure S1** showed that the mouse model of PTB was successfully constructed (**Supplementary Figures S1A, B**). Within 24 hours of the LPS/PBS injection, the mice were anesthetized with 1% pentobarbital/phenytoin immediately after the delivery of the first pup. Whole blood was collected using 5ml tubes (preloaded with 3ml TRIzol^®^ Reagent [Magen]) by removing the eyeball. Subsequently, the mice were dissected to obtain the placenta, uterus, decidua, and chorioamniotic membrane. The collected tissues were then placed in RNA*later* (Thermo Fisher Scientific) and stored at -80°C until they were ready to be used.

### RNA extraction and library preparation and sequencing

Total RNA was extracted from the mouse placenta, decidua, chorioamniotic membrane, uterus, and peripheral blood using TRIzol^®^ Reagent according to the manufacturer’s instructions (Magen). The quality and quantity of RNA were evaluated based on the A260/A280 absorbance ratio using a Nanodrop ND-2000 system (Thermo Scientific, USA), and the RNA integrity number (RIN) was determined using an Agilent Bioanalyzer 4150 system (Agilent Technologies, CA, USA). Only samples that met the quality criteria were selected for library construction. Paired-end libraries were prepared using an ABclonal mRNA-seq Lib Prep Kit (ABclonal, China) following the manufacturer’s instructions. Initially, mRNA was isolated from 1 μg of total RNA using oligo (dT) magnetic beads and fragmented using divalent cations at elevated temperatures in ABclonal First Strand Synthesis Reaction Buffer. Subsequently, first-strand cDNAs were synthesized using random hexamer primers and Reverse Transcriptase (RNase H) with mRNA fragments as templates, followed by second-strand cDNA synthesis using DNA polymerase I, RNAseH, buffer, and dNTPs. The synthesized double-stranded cDNA fragments were then adapter-ligated to prepare the paired-end library, and PCR amplification was performed on the adaptor-ligated cDNA. PCR products were purified using the AMPure XP system, and library quality was assessed using the Agilent Bioanalyzer 4150 system. Finally, the library preparations were sequenced on an Illumina Novaseq 6000 (or MGISEQ-T7) and 150 bp paired-end reads were generated.

### Metabolite extractions and LC-MS analysis

To extract metabolites from mouse placenta and uterus samples, 80 mg of the sample was mixed with 1 ml of cold extraction solvent (methanol/acetonitrile/H2O, 2:2:1, v/v/v) and thoroughly vortexed. The lysate was then homogenized using an MP homogenizer (24×2, 6.0 M/S, 60s, twice) and sonicated at 4°C (30min/once, twice) followed by centrifugation at 14,000g for 20 minutes at 4°C. The resulting supernatant was dried using a vacuum centrifuge at 4°C. For LC-MS analysis, the dried samples were re-dissolved in 100 μL of acetonitrile/water (1:1, v/v) solvent.

The extracts were analyzed using a Sciex TripleTOF 6600 quadrupole time-of-flight mass spectrometer coupled with hydrophilic interaction chromatography via electrospray ionization at Shanghai Applied Protein Technology Co., Ltd. The LC separation was performed on an ACQUIY UPLC BEH Amide column (2.1 mm × 100 mm, 1.7 µm particle size, Waters, Ireland) using a gradient of solvent A (25 mM ammonium acetate and 25 mM ammonium hydroxide in water) and solvent B (acetonitrile). The gradient started with 95% B for 1 minute and was linearly decreased to 65% B over 11 minutes, then reduced to 40% B in 0.1 minutes and maintained for 4 minutes. Finally, the gradient was increased to 95% B in 0.1 minutes, and a 5-minute re-equilibration period was employed. The flow rate was 0.5 mL/minute, and the column temperature was maintained at 25°C. The auto-sampler was kept at 4°C, and the injection volume was 2 µL.

The mass spectrometer was operated in both negative and positive ionization modes. The ESI source conditions were set as follows: Ion Source Gas1 (Gas1) at 60, Ion Source Gas2 (Gas2) at 60, Curtain Gas (CUR) at 30, source temperature at 600°C, and IonSpray Voltage Floating (ISVF) at ±5500V. For MS acquisition, the instrument was set to acquire over the m/z range of 60-1000 Da, and the accumulation time for the TOF MS scan was set at 0.20s/spectra. In auto MS/MS acquisition, the instrument was set to acquire over the m/z range of 25-1000 Da, and the accumulation time for product ion scan was set at 0.05s/spectra. The product ion scan was obtained using information-dependent acquisition (IDA) with high sensitivity mode selected. The following parameters were used: collision energy (CE) was fixed at 35V with ±15eV; declustering potential (DP) at 60V (+) and −60V (-); exclude isotopes within 4Da, and candidate ions to monitor per cycle were set at 10.

### Real-time quantitative PCR validation analysis

To validate the transcriptome sequencing outcomes, RT-qPCR was performed. RNA samples were first reverse transcribed to cDNA using the PrimeScript RT Reagent kit (Takara, Japan) following the manufacturer’s instructions. qPCR was carried out using TB Green qPCR Master Mix (Takara, Japan) on a Q5 real-time PCR System (Life Technology, USA). The following thermocycling conditions were used for the PCR: an initial denaturation at 95°C for 30 seconds, 40 cycles of 95°C for 5 seconds, and 60°C for 34 seconds, followed by a final extension at 95°C for 15 seconds, 60°C for 1 minute, and 95°C for 15 seconds. The relative expression of target genes was calculated using the 2^-ΔΔ^Ct method, with ACTB as the internal reference gene.

### Bioinformatics analysis

The transcriptomic data generated from the Illumina platform were subjected to bioinformatics analysis using an in-house pipeline developed by Shanghai Applied Protein Technology. After quality control, the clean reads were aligned separately to the reference genome in orientation mode using HISAT2 software (http://daehwankimlab.github.io/hisat2/) to obtain mapped reads. The reads numbers mapped to each gene were counted using FeatureCounts (http://subread.sourceforge.net/), and the FPKM of each gene was calculated based on the gene length and read count mapped to it. Differential expression genes (DEGs) analysis was carried out using the DESeq2 package (http://bioconductor.org/packages/release/bioc/html/DESeq2.html). DEGs with |log2FC| > 1 and *P* adj < 0.05 were considered significantly differentially expressed genes. Transcription factor (TF) analysis of DEGs was extracted directly from the AnimalTFDB (http://bioinfo.life.hust.edu.cn/AnimalTFDB/) and PlantTFDB database (http://planttfdb.cbi.pku.edu.cn/). Protein-protein interaction (PPI) analysis was performed based on the known and predicted interactions in the STRING database (https://www.string-db.org/). Finally, alternative splicing (AS) analysis was conducted using the rMATS software (http://rnaseq-mats.sourceforge.net/index.html).

The metabolomic raw MS data (wiff.scan files) were first converted to MzXML files using ProteoWizard MSConvert and then imported into the freely available XCMS software. For peak picking, the following parameters were used: centWave m/z = 25ppm, peakwidth = c (10, 60), prefilter = c (10, 100). Peak grouping was conducted with bw = 5, mzwid = 0.025, and minfrac = 0.5. In the extracted ion features, only variables with more than 50% of the nonzero measurement values in at least one group were retained. Compound identification of metabolites was performed by matching MS/MS spectra with an in-house database established with available authentic standards. After normalization to total peak intensity, the processed data were uploaded into SIMCA-P (version 14.1, Umetrics, Umea, Sweden) for multivariate data analysis, including Pareto-scaled principal component analysis (PCA) and orthogonal partial least-squares discriminant analysis (OPLS-DA). The robustness of the model was evaluated using 7-fold cross-validation and response permutation testing. The contribution of each variable to the classification was determined by calculating its variable importance in the projection (VIP) value in the OPLS-DA model. Statistical significance was determined using an unpaired Student’s *t*-test, where VIP value >1 and *P* < 0.05 were considered statistically significant.

Transcriptomic GO function enrichment and KEGG pathway enrichment analyses were performed using the clusterProfiler R software package. For metabolomic KEGG pathway annotation, the metabolites were blasted against the online Kyoto Encyclopedia of Genes and Genomes (KEGG) database to retrieve their COs, which were then mapped to pathways in KEGG. KEGG pathway enrichment analyses were conducted using Fisher’s exact test, with the whole metabolites of each pathway considered as the background dataset. GO or KEGG functions were considered significantly enriched when *P* < 0.05.

### Integrated network analysis of the transcriptome and metabolome

The Spearman statistical method was employed to analyze the correlation coefficient between the significant differential genes and metabolites. The resulting correlation matrix was visualized using R and Cytoscape (v3.8.0) software, which allowed exploration of the interaction between genes and metabolites from multiple perspectives, including matrix heat maps, hierarchical clustering, and correlation networks. Spearman correlation network analysis was performed on genes and metabolites with significant correlation coefficient values |r| ≥ 0.5 and *P* < 0.01.

### Data analysis

Statistical analyses were performed using GraphPad Prism 9 software. An independent samples *t*-test or Fisher’s exact test was used to compare groups, as appropriate. Adjusted *P*-values were calculated using the Benjamani-Hochberg false discovery rate (FDR), with an FDR value ≤ 0.01 considered significant. A *P*-value < 0.05 was considered statistically significant.

## Results

### Identification of differentially expressed genes and metabolites

**Figure 1:**
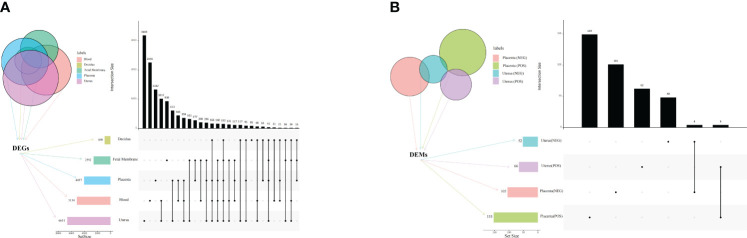
Venn diagram and UpSet plot of DEGs and DEMs. **(A)** Venn diagram and UpSet plot showing the shared and unique DEGs among mouse placenta, decidua, chorioamniotic membrane, uterus and peripheral blood (*P* < 0.05, PBS vs. LPS). **(B)** Venn diagram and UpSet plot showing the shared and unique DEMs among mouse placenta and uterus (*P* < 0.05, PBS vs. LPS). The horizontal bars display the number of DEGs of different tissues, while the vertical bars show the size of genes identified by only 1 comparison and the intersection sets. Single dots and the corresponding horizontal bars represent the number of genes unique to the data set and not shared between the other comparisons. The UpSet plot was performed using the OmicStudio tools at https://www.omicstudio.cn/tool. DEGs, differentially expressed genes; DEMs, differentially expressed metabolites.

Principal component analysis (PCA) revealed a clear distinction between the two treatments (LPS vs. PBS) in the placenta, chorioamniotic membrane, decidua, uterus, and peripheral blood samples (**Figures S2A–E**). Besides, all raw data of the samples passed quality control (QC) (**Supplementary Table S1**). A total of 497, 2133, 522, 1549, and 2579 DEGs (*P* adj < 0.05 and |log2(FC)| > 1) were identified in placenta (LPS vs. PBS), chorioamniotic membrane (LPS vs. PBS), decidua (LPS vs. PBS), uterus (LPS vs. PBS), and peripheral blood (LPS vs. PBS), respectively (**Supplementary Table S2**). The volcano plot of gene expression levels illustrates the distribution of up- and down-regulated genes in different tissues (**Supplementary Figures S3A–E**). Comparison of the placenta, chorioamniotic membrane, decidua, uterus, and peripheral blood revealed 152 significant overlapping differential genes (*P* < 0.05) (**Figure 1A**; **Table 1**; **Supplementary Table S3**).

**Table 1:** Common DEGs and DEMs in the different tissues.

Tissues	Placenta	Chorioamniotic Membrane	Decidua	Uterus	Peripheral Blood
Genes	JUNB,SOCS3,TRIM21,STAT3,TLR3,PML,ADORA2B,RNF114,LITAF,HAP1,TAP1,DAXX,IFIH1,DHX58,WHAMM,MX2,OAS1B,TOR3A,PARP10,ACOD1,OAS1A,MLKL,USP18,CD274,IFI205,IER3,MYD88,IGTP,OAS1G,NOD2,SMOX,RASSF2,NMI,TNFRSF1A,PSMB9,CCL3,CCL4,IFIT2,TNF,TIMP1,GBP3,SOCS1,CLEC4E,CCL2,CXCL1,OASL2,MITD1,MARCKS,HCAR2,ICAM1,OGFR,RELB,TNIP1,CSNK1A1,IRF1,CASP4,IL1B,SNX10,CCL7,RSAD2,SELP,ZBP1,GM4951,PTX3,NFKBIA,SLFN4,GM12250,BHLHE40,PFKFB3,OLFR56,CRISPLD2,LILR4B,CD14,HERC6,RND1,TRIM30D,XAF1,MS4A4C,IRF7,CCRL2,BCL2A1D,TRIM30A,IL6,LCN2,NFIL3,TNFAIP2,ZFP36,PSME2,RTP4,PSME2B,FPR2,SAT1,CD47,H2Q4,RNF19B,IRGM1,HCK,MXD1,PIK3R5,IFI209,EIF6,GBP6,BCL3,CCL12,IFITM3,PSMB8,MEFV,NFKBIB,TIFA,PHF11B,SOD2,LCP2,WFDC17,BCL2A1A,TLR2,NFKBIE,SLFN8,PSMB10,HP,FCGR4,IFI44,NOS2,GBP9,ACOT2,SP140,MAP3K8,OAS3,SLFN2,CD300LF,MS4A6D,GLIPR2,PHF11D,TNFRSF1B,MNDAL,EPSTI1,FCGR2B,GM4841,PNRC1,AA467197,IL1F9,IL15RA,CXCL16,PNP,RHBDF2,UBE2L6,APOBEC3,SLCO3A1,GPR84,BATF2,KIFC2,IFITM1,GBP4				
Tissues	Placenta (POS)			Uterus (POS)	
Metabolites	Nicotinamide N-oxide, N-acetyl-d-lactosamine, His-Asp, 5,8-dimethylquinolin-4-ol				
Tissues	Placenta (NEG)			Uterus (NEG)	
Metabolites	L-dihydroorotate, Trans-3’-hydroxycotinine o-.beta.-d-glucuronide, Humulone, Pentobarbital				

DEGs, differentially expressed genes; DEMs, differentially expressed metabolites; POS, positive; NEG, negative.

**Figure 2:**
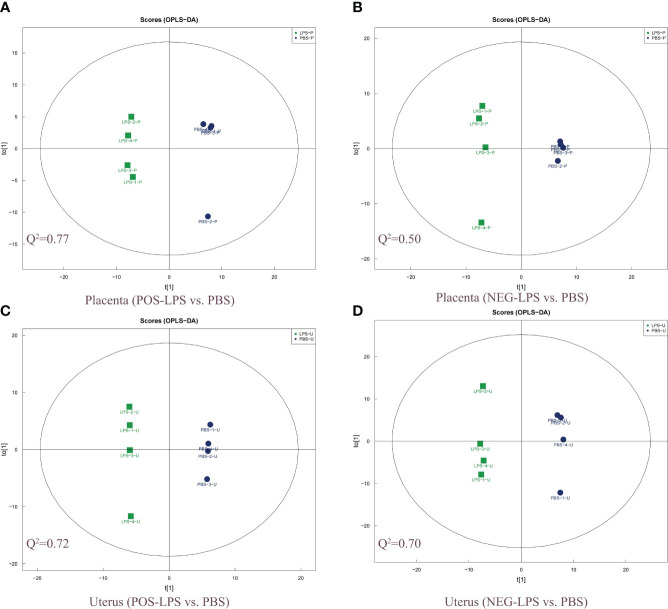
Two-dimensional scatter score plot of OPLS-DA. Scatter score plot of OPLS-DA in the placenta for **(A)** POS-LPS vs. PBS and **(B)** NEG-LPS vs. PBS, and the uterus for **(C)** POS-LPS vs. PBS and **(D)** NEG-LPS vs. PBS. An OPLS-DA model was generated using a seven-fold cross-validation procedure, with the model evaluation parameters (R^2^Y, Q^2^) obtained by 7-fold cross-validation. Here, Q^2^ > 0.5 indicates that the model is stable and reliable, 0.3 < Q^2^ ≤ 0.5 indicates that the model is stable, and Q^2^ < 0.3 indicates that the model has low reliability. The OPLS-DA analysis was performed in both the positive and negative ion modes. OPLS-DA, orthogonal partial least squares discriminant analysis; POS, positive; NEG, negative.

Orthogonal partial least squares discriminant analysis (OPLS-DA) revealed significant differences between the two treatment groups (LPS vs. PBS) in the placenta and uterus in both positive and negative ion modes (**Figures 2A–D**). A permutation test was conducted to ensure model validity and stability and to prevent over-fitting (**Supplementary Figures S4A–D**). In total, 114 and 54 differentially expressed genes and metabolites (DEMs) were identified in the placenta in positive and negative ion modes, respectively, while 158 and 146 DEMs were identified in the uterus in positive and negative ion modes, respectively (**Supplementary Table S4**), all with OPLS-DA VIP>1 and *P* value < 0.05. When comparing the placenta with the uterus, a total of 8 significant overlapping differential genes (*P* < 0.05) were identified in both positive and negative ion modes (**Figure 1B**; **Table 1**).

### KEGG and GO functional enrichment analyses of DEGs

**Figure 3:**
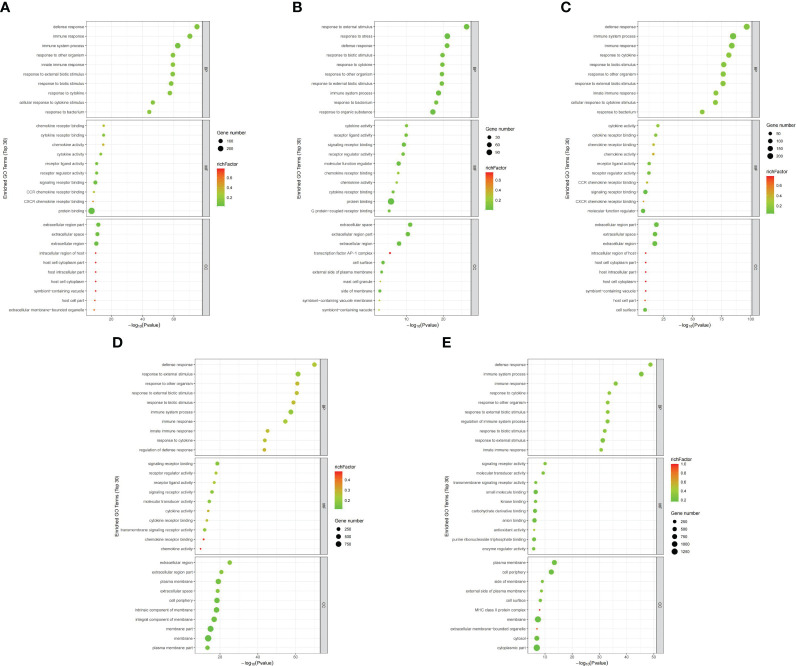
Differential gene GO annotation visualization (top 30). Bubble chart for the **(A)** placenta (LPS vs. PBS), **(B)** chorioamniotic membrane (LPS vs. PBS), **(C)** decidua (LPS vs. PBS), **(D)** uterus (LPS vs. PBS), and **(E)** peripheral blood (LPS vs. PBS). The annotated GO term is arranged from small to large according to *log10(P value)* (abscissa), and the ordinate represents specific functional description information. GO includes molecular function (MF), biological process (BP) and cellular component (CC) (red). Bubble size represents the number of DEGs (*P* adj < 0.05, |log2(fold change)| > 1) involved in the GO enrichment. The rich factor was calculated as follows: (number of DEGs in GO term)/(number of genes contained in GO term).

GO functional analysis revealed that several GO terms were common among placenta (LPS vs. PBS), chorioamniotic membrane (LPS vs. PBS), decidua (LPS vs. PBS), uterus (LPS vs. PBS) and peripheral blood (LPS vs. PBS). These shared GO terms included biological process (BP) terms such as response to cytokine (GO:0034097), defense response (GO:0006952) and response to biotic stimulus (GO:0009607), molecular function (MF) terms such as signaling receptor binding (GO:0005102), cytokine activity (GO:0005125) and chemokine activity (GO:0008009), and cellular component (CC) terms such as extracellular region (GO:0005576) and cell surface (GO:0009986) in cellular component (CC) (**Figures 3A–E**; **Supplementary Table 5**).

**Figure 4:**
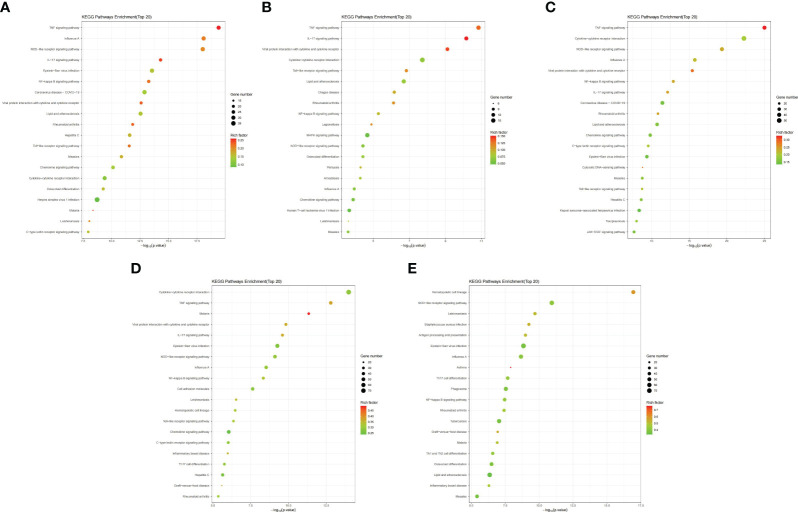
Bubble maps of KEGG pathway enrichment of DEGs. Bubble maps for the **(A)** placenta (LPS vs. PBS), **(B)** chorioamniotic membrane (LPS vs. PBS), **(C)** decidua (LPS vs. PBS), **(D)** uterus (LPS vs. PBS), and **(E)** peripheral blood (LPS vs. PBS). In this diagram, the degree of KEGG enrichment was measured using the Rich factor, *P*-value and the number of genes enriched in a given pathway. Rich factor refers to the ratio of the number of DEGs in the pathway to the total number of genes. *P* value refers to the significance of pathway enrichment, and the value range is [0,1]. The closer it is to zero, the more significant the enrichment is. The bubble size represents the number of DEGs (*P* adj < 0.05, |log2(fold change)| > 1) involved in the KEGG enrichment. Each point represents a KEGG pathway, the ordinate represents the pathway name, and the abscissa represents the log10(*P* value). The Rich factor is represented by color, with a darker red color indicating a higher significance level.

The top 20 enriched pathways were identified through KEGG functional enrichment analysis of the DEGs in different mouse tissues (**Figures 4A–E**; **Supplementary Table S5**). Further analysis showed that the NOD-like receptor signaling pathway (mmu04621), TNF signaling pathway (mmu04668), IL-17 signaling pathway (mmu04657) and NF-kappa B signaling pathway (mmu04064) were significantly enriched in all tissues or maternal-fetal interfaces (placenta, chorioamniotic membrane, decidua, and uterus) (**Figures 4A–E**). Besides, KEGG functional analysis of the 152 significant overlapping DEGs (*P* < 0.05) showed similar results (**Supplementary Table S6**).

### Protein-protein interaction network

**Figure 5:**
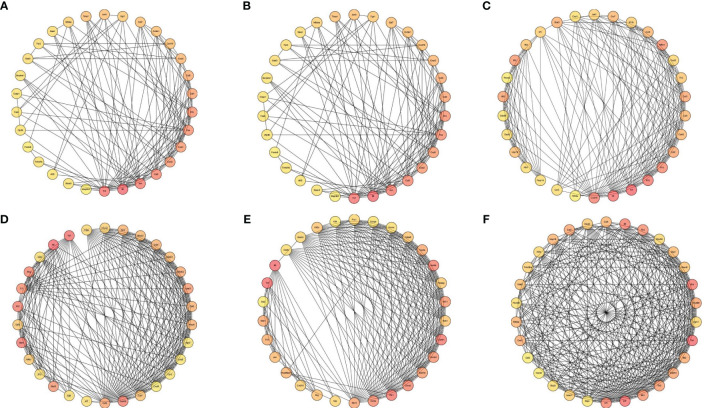
Interaction network diagram of DEGs (Top 30). Interaction network diagram for the **(A)** placenta (LPS vs. PBS), **(B)** chorioamniotic membrane (LPS vs. PBS), **(C)** decidua (LPS vs. PBS), **(D)** uterus (LPS vs. PBS), **(E)** peripheral blood (LPS vs. PBS), and **(F)** co-expressed DEGs in the placenta (LPS vs. PBS), chorioamniotic membrane (LPS vs. PBS), decidua (LPS vs. PBS), uterus (LPS vs. PBS) and peripheral blood (LPS vs. PBS) (*P* adj < 0.05, |log2(fold change)| > 1). Top 30 hub genes identified using CytoHubba. Each circle in the figure represents a gene, and the lines between circles represent interactions between genes. The color of a circle reflects the number of interactions that the corresponding gene has, with a darker color indicating a higher degree of connectivity.

The PPI network was constructed using the online STRING database, and further analysis was conducted using Cytoscape software. CytoHubba was employed to screen for key genes, and all molecular nodes and edges were calculated. The top 30 key genes were obtained using the multiscale curvature classification algorithm. The interactions of the top 30 DEGs in the placenta, chorioamniotic membrane, decidua, uterus and peripheral blood, as well as the 152 significant overlapping DEGs (*P* < 0.05), were explored through PPI analysis (**Figures 5A–F**).

### GSEA, TF and AS analysis of DEGs

**Figure 6:**
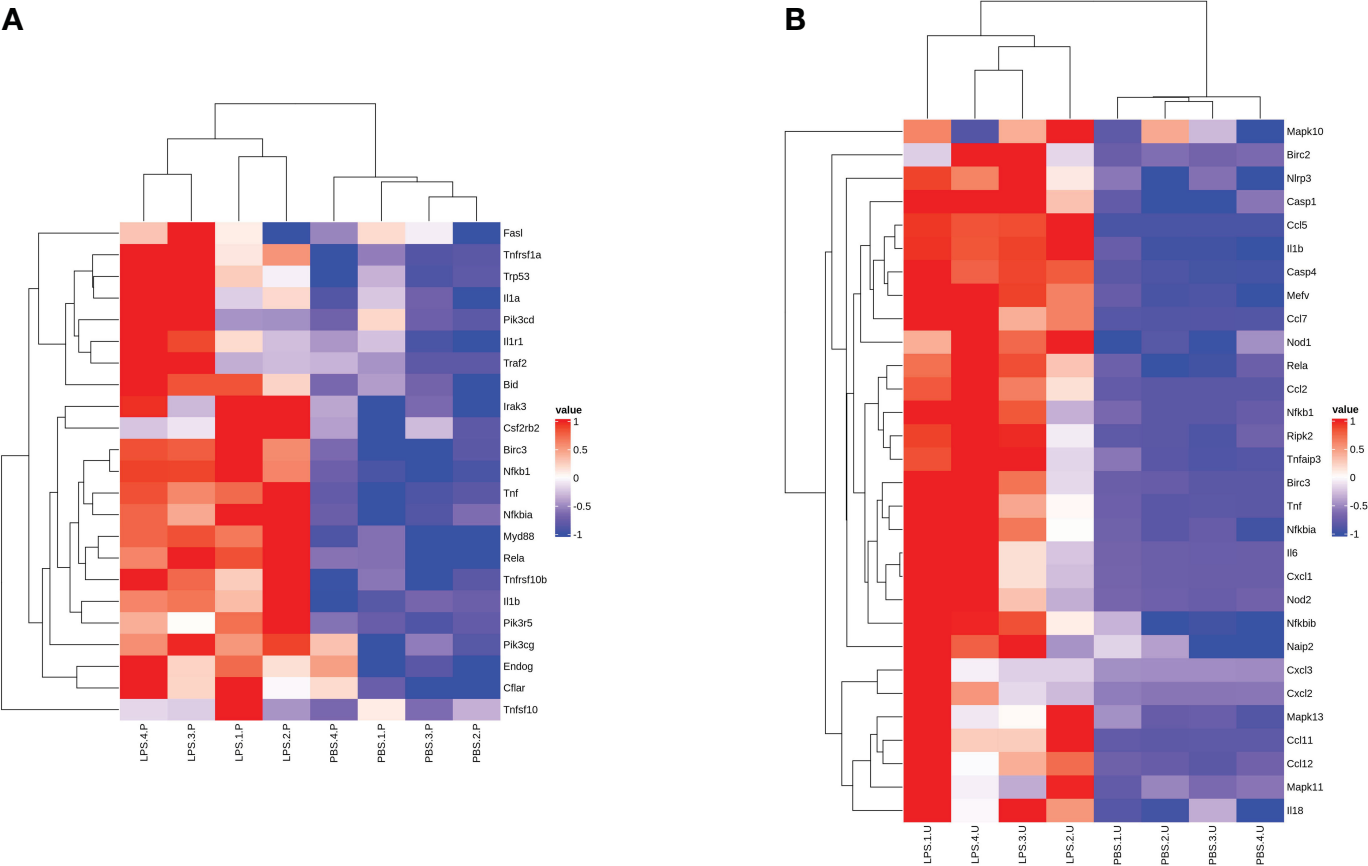
Gene expression heat map of significant GSEA pathway. Heat map for the **(A)** placenta (LPS vs. PBS) and **(B)** uterus (LPS vs. PBS) showing the clustered list of genes according to significant functions detected by GSEA with KEGG pathways. The figure uses a color scheme to represent the expression levels of protein-coding genes, with red indicating relatively high expression and blue indicating relatively low expression. GSEA, gene set enrichment analysis.

Gene Set Enrichment Analysis (GSEA, http://software.broadinstitute.org/gsea), TF analysis (TFscan software), and alternative splicing (AS) events analysis were used for further analysis of the DEGs. GSEA showed the KEGG Apoptosis pathway in the placenta, while the NOD-like receptor signaling pathway was significantly enriched in the chorioamniotic membrane, decidua, uterus and peripheral blood (**Supplementary Figures S5A–E**). The results of cluster analysis of all DEGs in the apoptosis pathway and NOD-like receptor signaling pathway are depicted in heat maps (**Figures 6A, B**). The TFs regulating DEGs caused by LPS in different mouse tissues were found to be different (**Supplementary Figures S6A–E**; **Table S5**). AS analysis using rMATS software (http://rnaseq-mats.sourceforge.net/index.html) revealed that the AS patterns induced by LPS in different mouse tissues differed (**Supplementary Figures S7A–E**). AS enrichment (top 20) showed that Spliceosome, Notch signaling pathway, and Mitophagy pathway were the most significantly enriched (**Supplementary Figures S8A–E**).

### Validation of gene expression of the differentially expressed mRNAs by qRT-PCR.

**Figure 7:**
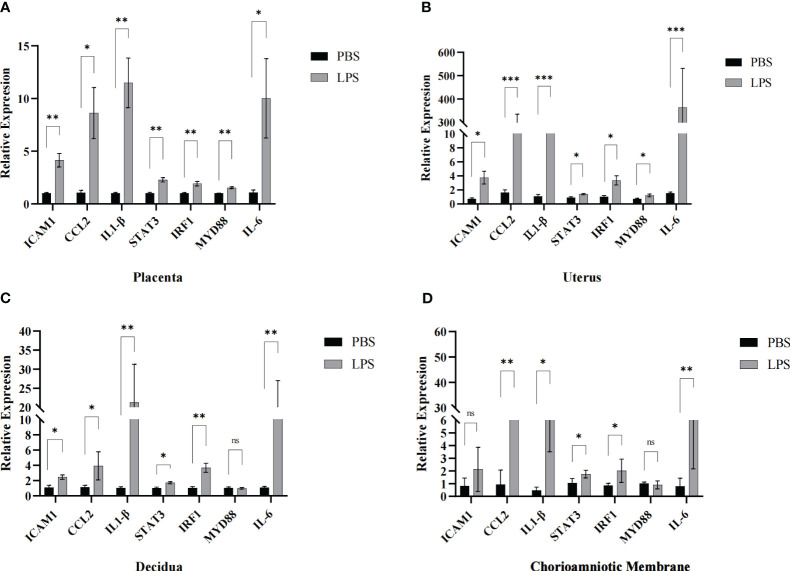
Validation of maternal-fetal interface DEGs. The transcriptomic results of ICAM1, CCL2, IL1-β, STAT3, IRF1, MYD88 and IL-6 were further confirmed by qPCR in the **(A)** placenta, **(B)** uterus, **(C)** decidua and **(D)** chorioamniotic membrane respectively (LPS vs. PBS, n=4 respectively). The expression trends of candidate mRNAs were consistent with the sequencing results. Data are presented as 2^−ΔΔ^ct values (mean ± SEM). *P < 0.05, **P < 0.01 and ***P < 0.001; ns, not significant.

To verify the accuracy of the RNA sequencing results, seven mRNA transcripts were randomly selected from the 152 significant overlapping DEGs (*P* < 0.05) and validated through qRT-PCR. Total RNA was extracted from the mouse maternal-fetal interface (placenta, chorioamniotic membrane, decidua and uterus) with LPS or PBS treatment, and cDNA was synthesized. The expression levels of the seven mRNAs were measured using TB Green qPCR Master Mix (Takara, Japan). The results indicated that the mRNA levels of ICAM1, CCL2, IL-1β, IL-6, STAT3, and IRF1 were significantly upregulated in the placenta, chorioamniotic membrane, decidua, and uterus, which was consistent with the RNA-seq data (**Figures 7A–D**). However, there was no significant difference in the expression of MYD88 in the chorioamniotic membrane and decidua, which was inconsistent with the RNA-seq results (**Figures 7C, D**). The primer sequences used in the qPCR are listed in **Table 2**.

**Table 2:** Sequences of the primers used in qRT-PCR experiments.

mRNAs	Primer	Sequence(5’ to 3’)
ICAM1	Forward	GTGATGCTCAGGTATCCATCCA
	Reverse	CACAGTTCTCAAAGCACAGCG
CCL2	Forward	TTAAAAACCTGGATCGGAACCAA
	Reverse	GCATTAGCTTCAGATTTACGGGT
IL-1β	Forward	GCAACTGTTCCTGAACTCAACT
	Reverse	ATCTTTTGGGGTCCGTCAACT
IL-6	Forward	TAGTCCTTCCTACCCCAATTTCC
	Reverse	TTGGTCCTTAGCCACTCCTTC
STAT3	Forward	CAATACCATTGACCTGCCGAT
	Reverse	GAGCGACTCAAACTGCCCT
MYD88	Forward	TCATGTTCTCCATACCCTTGGT
	Reverse	AAACTGCGAGTGGGGTCAG
IRF1	Forward	ATGCCAATCACTCGAATGCG
	Reverse	TTGTATCGGCCTGTGTGAATG
ACTB	Forward	GGCTGTATTCCCCTCCATCG
	Reverse	CCAGTTGGTAACAATGCCATGT

qPCR, real-time fluorescent quantitative PCR.

### The proportions of distinct immune cell subpopulations

**Figure 8:**
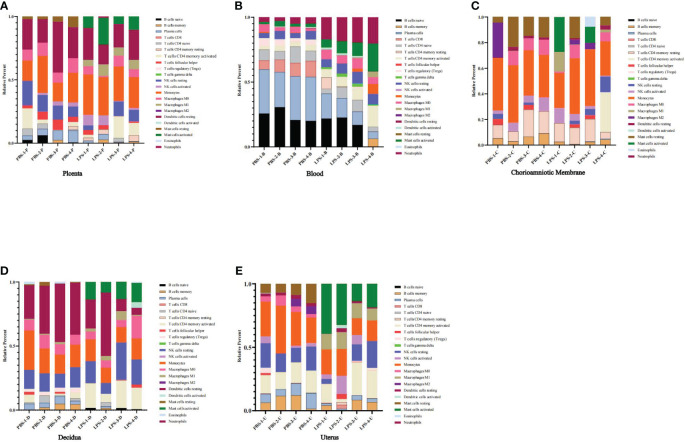
The proportions of distinct immune cell subpopulations showed by CIBERSORT. **(A)** Placenta (LPS vs. PBS). **(B)** Peripheral blood (LPS vs. PBS). **(C)** Chorioamniotic membrane (LPS vs. PBS). **(D)** Decidua (LPS vs. PBS). **(E)** Uterus (LPS vs. PBS). Histogram showing the CIBERSORT estimation of immune cell infiltration based on the LM22 file. Different colors represent different types of immune cells.

To investigate immune cell infiltration in different mouse tissues (placenta, chorioamniotic membrane, decidua, and uterus), we analyzed 22 immune cell phenotypes in the RNA-seq data of the CIBERSORT LM22 file. The analysis revealed that LPS treatment increased the proportion of monocytes in the placenta, mast cells activated, dendritic cells resting, macrophages M0, and NK cells resting in blood; NK cells resting in chorioamniotic membrane; mast cells activated and T cells CD4 memory activated in decidua; and NK cells, mast cells activated, macrophages M1, and T cells CD4 memory activated in the uterus (**Figures 8A–E**).

### Placenta and uterus metabolomics analysis

**Figure 9:**
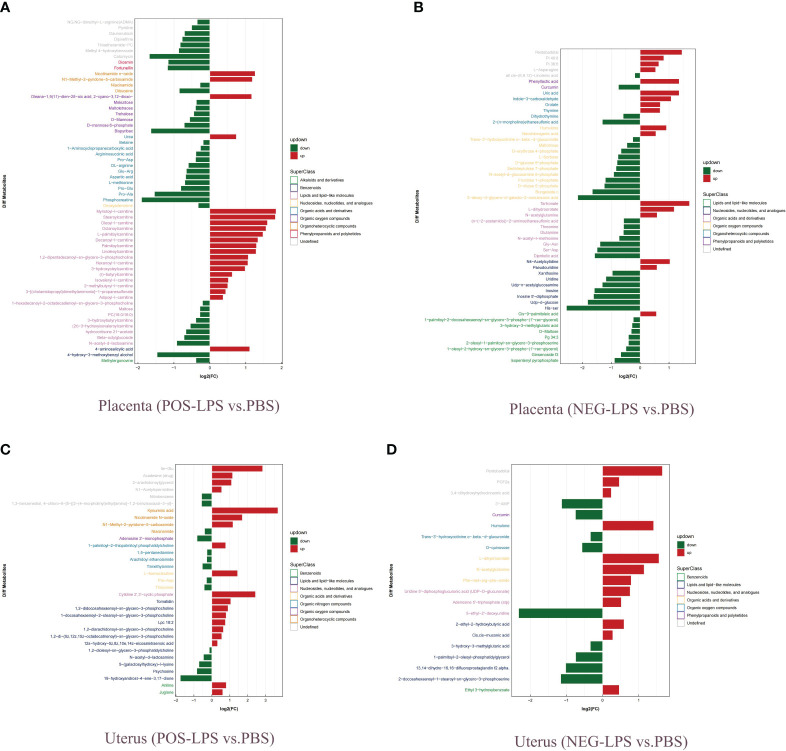
Analysis of fold change of DEMs. **(A)** Placenta (POS-LPS vs. PBS). **(B)** Placenta (NEG-LPS vs. PBS). **(C)** Uterus (POS-LPS vs. PBS). **(D)** Uterus (NEG-LPS vs. PBS). DEMs were examined by OPLS-DA, and variable importance in projection (VIP > 1) and t-test (*P* < 0.05) were used for screening. Green represents downregulated DEMS, and red represents upregulated DEMS. Different colors of ordinate represent different superfamilies of DEMs. OPLS-DA, orthogonal partial least squares discriminant analysis; VIP, variable importance for the projection; POS, positive; NEG, negative; DEMs, differentially expressed metabolites.

Histogram and heatmap analysis of the differentially expressed metabolites (DEMs) showed significant changes in metabolites in mouse placenta and uterus after LPS intervention, including Curcumin, Humulone, L-dihydroorotate, PGF2α, and Nicotinamide N-oxide (**Supplementary Figures S9**, **S10A–D**). Further analysis showed that the DEMs mainly belonged to organic oxygen compounds, lipids and lipid-like molecules, organic acids and derivatives, and organic nitrogen compounds (**Figures 9A–D**). Additionally, a combined analysis of DEMs in the placenta and uterus found that Nicotinamide N-oxide, N-acetyl-d-lactosamine, His-Asp, 5,8-dimethylquinolin-4-ol, L-dihydroorotate, Trans-3’-hydroxycotinine o-.beta.-d-glucuronide, Humulone, and Pentobarbital showed significant changes in both mouse placenta and uterus after LPS intervention (**Table 1**; **Supplementary Table S4**).

KEGG enrichment analysis found that Thyroid hormone synthesis, Lysosome, NOD-like receptor signaling pathway and Protein digest and absorption pathway were significantly enriched (**Supplementary Figure S11A, B**). In addition, molecular interaction network analysis was performed using the DEMs (OPLS-DA VIP>1 and *P* value < 0.05) uploaded to the online STRING database, and Cytoscape software was used for further analysis. CytoHubba was utilized to screen for the key metabolites, and the results showed that the interaction of key metabolites in the placenta was more obvious (**Supplementary Figures S12A–D**).

### Integrative analysis of the metabolome and transcriptome

**Figure 10:**
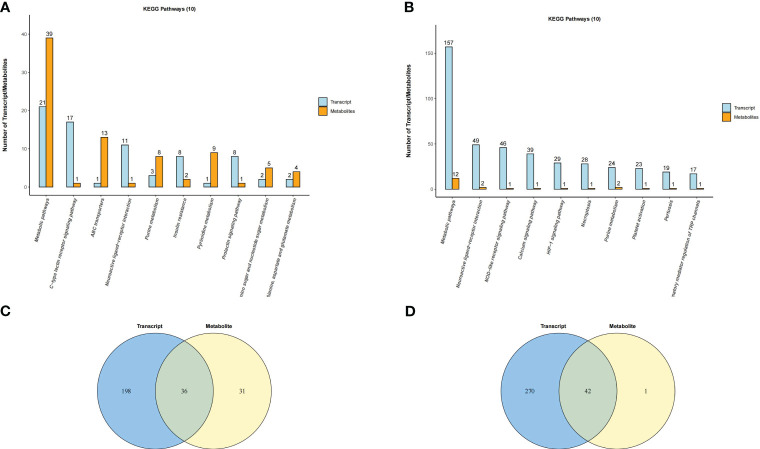
Venn diagram and histogram of pathways involved by both DEGs and DEMs. **(A)** Placenta (LPS vs. PBS) and **(B)** Uterus (LPS vs. PBS). Venn diagram of pathways in which the DEGs and DEMs are involved. Different color circles represent different omics, in which blue represents transcriptomic and yellow represents metabolomic; the values in the circle represent the common/unique pathways of the two omics. **(C)** Placenta (LPS vs. PBS) and **(D)** Uterus (LPS vs. PBS). Histogram of pathways in which the DEGs and DEMs are involved (TOP10). Each column in the figure represents a KEGG pathway, with different colors representing different omics, among which blue represents transcriptomic, and orange represents metabolomic. The number is ranked from high to low. The higher the column, the more DEGs or DEMs the biological pathway was annotated.

DEGs and DEMs with significantly altered levels (*P* adj < 0.05 and |log2(FC)| > 1 and OPLS-DA VIP>1 and *P* value < 0.05) were analyzed, and Spearman correlation analysis revealed a significant correlation between them (**Supplementary Figures S13A, B**; **Table S7**). KEGG pathway analysis showed that DEGs and DEMs were both involved in the Insulin resistance pathway in the placenta and the NOD-like receptor signaling pathway in the uterus (**Figures 10A, B**; **Supplementary Figures S14**, **S15A, B**). In addition, DEGs and DEMs commonly participated in the Prolactin signaling pathway, Ovarian steroidogenesis, and Calcium signaling pathway (**Supplementary Figures S15C, D**). Overall, there were 36 and 42 common KEGG pathways of DEGs and DEMs in the uterus and placenta, respectively (**Figures 10C, D**). Correlation network analysis was performed to assess potential interactions between DEGs and DEMs with LPS intervention, and the results showed that differential inflammatory-related factors (IL1-β and NLRP3) or chemokines (CCL3) interacted with DEMs (**Supplementary Figures S16A, B**).

## Discussion

In the present study, we comprehensively explored the tissue-dependent effects of acute exposure to LPS via transcriptomics and metabolomics analysis in mice placenta, chorioamniotic membrane, decidua, uterus and peripheral blood. The results revealed a total of 152 common DEGs (*P* < 0.05) in mice placenta, chorioamniotic membrane, decidua, uterus and peripheral blood, while 8 common DEMs (*P* < 0.05) were found in mouse placenta and uterus after LPS exposure. We further investigated the pathways enriched in these common DEGs and DEMs and found that they were both enriched in the NOD-like receptor signaling pathway, while apoptosis, oxidative phosphorylation and hormone synthesis pathways showed tissue-specific correlations after LPS intervention. Additionally, we identified interactions between DEGs and DEMs, suggesting their combined role in the LPS-induced PTB model of mice. We also observed alterations in upstream regulators and master regulators important for inflammatory reactions, such as STAT1, STAT3, and NFKB1, in LPS-exposed tissues.

PTB is a multifaceted syndrome with various potential underlying pathophysiologic mechanisms. Increased uterine contractility, cervical ripening and rupture of chorioamniotic membranes are the common pathways of both TB and PTB (3, 14, 23). Placental insufficiency, including conditions such as placental abruption, chronic chorioamnionitis and twin pregnancy, appears to be responsible for many PTB cases, emphasizing the placenta’s significant role in PTB pathogenesis (24). In a study by Lien et al., metabolomics and transcriptomics were combined to demonstrate that intrauterine inflammation can lead to placental dysfunction in mice by altering mitochondrial function and energy metabolism (25). Similarly, our study showed significant changes in transcriptomics and metabolomics profiles in mice with an intraperitoneal injection of LPS. Apart from the inflammation-related NF-kappa B signaling pathway and TNF signaling pathway, we also observed significant changes in the apoptosis pathway, metabolic pathway and oxidative phosphorylation pathway, suggesting that inflammation may lead to placental dysfunction by promoting placental cell apoptosis, increasing placental oxidative stress and metabolic disorders. Mitochondrial dysfunction, caused by increased oxidative stress and inflammatory response, is a key factor in the pathophysiology of human pregnancy, including PTB, which is strongly associated with placental tissue rich in mitochondria (26, 27).

Uterine contraction is a defining characteristic of labor, and most drugs, such as progesterone, betamimetics, calcium channel blockers, magnesium sulfate, oxytocin receptor antagonists, nitric oxide donors, and others, used in clinical practice aim to inhibit uterine contraction. However, these drugs have limitations in terms of safety and clinical application (28, 29). Humans and animals (i.e., mice) share a high degree of similarity in terms of uterine contraction. Through transcriptomic and metabolomic analysis, we found significant changes in the cytokine-cytokine receptor interaction, calcium signaling pathway, metabolic pathways and steroid hormone biosynthesis pathway in the mice uterus following intraperitoneal injection of LPS. Additionally, activation of the decidua is an important feature of both the TB and PTB process (30). The decidua contains a large number of immune cells that can produce cytokines. While the number of decidual macrophages increased in both TB and PTB, the number of neutrophils increased significantly only in patients with PTB (10). Prostaglandin E2 and F2 are also mainly synthesized in the decidua and are essential in promoting uterine contraction (31). Our study found that intraperitoneal injection of LPS can cause decidual activation. The TNF signaling pathway, NOD-like receptor signaling pathway, Cytokine-cytokine receptor interaction, and Chemokine signaling pathway were significantly enriched. In addition to the decidua, the chorioamniotic membrane also plays a crucial role in TB or PTB, particularly during preterm premature rupture of membranes (PPROM) (3). Studies have shown that both infectious and sterile inflammation can cause rupture of the chorioamniotic membrane and lead to PTB (32). Moreover, chorioamniotic membrane senescence is also considered one of the possible signals for the onset of labor (33). Although our study found that the chorioamniotic membrane was least affected by intraperitoneal injection of LPS, with only 898 DEGs (*P* < 0.05), most of these DEGs were related to inflammatory response, including the TNF signaling pathway and NF-kappa B signaling pathway.

Our study confirmed that inflammation induced by intraperitoneal injection of LPS could lead to PTB in mice. Several inflammatory factors, such as IL1β and IL6, as well as chemokines, such as CXCL1 and CCL2, exhibited significant differences in various tissues. Additionally, some transcription factors, such as IRF1 and STAT3, showed similar changes. Notably, the significance of the identified 152 co-expressed DEGs in the placenta, chorioamniotic membrane, decidua, uterus and peripheral blood requires further investigation. Meanwhile, the involvement of genes, such as NLRP3, in the common enriched NOD-like receptor signaling pathway and their role in PTB has been previously reported (34). Furthermore, our study found that intraperitoneal injection of LPS in mice led to significantly different changes in placental and uterine metabolites. However, several metabolites, including Nicotinamide N-oxide, N-acetyl-d-lactosamine, His-Asp, 5,8-dimethylquinolin-4-ol, L-dihydroorotate, Trans-3’-hydroxycotinine o-.beta.-d-glucuronide, Humulone and Pentobarbital, showed similar changes. Nicotinamide N-oxide, a major nicotinamide catabolite in mice, may help inhibit the excessive production of inflammatory cytokines (35). N-acetyl-d-lactosamine is a Galectin-3 inhibitor that plays a role in immunosuppression mediated by Gal-3 and prostate cancer stem cell-like cells (36). On the other hand, His-Asp is a downstream product of the cytokine signaling pathway, which may regulate the function of genes associated with plant cytokine signaling pathway (37). L-dihydroorotate is an intermediate product of pyrimidine metabolism and a substrate of dihydroorotate dehydrogenase in mitochondria. Interestingly, Humulone can inhibit COX-2 expression through the NF-kappa B pathway and A-P1 pathway after drug-induced mouse skin damage (38). Metabolomics can detect the processes that have occurred and help us to further investigate our findings.

Although the PTB model induced by intraperitoneal injection of LPS in late pregnancy of mice is a widely used method in research (19, 39, 40), there were limitations to this study that should be acknowledged. First, our experiment began after the first neonatal mouse was delivered, and although the interval time was shorter than 3 hours, we cannot rule out the effect of LPS action time on our experimental results. Second, considering that mice are multiparous, different pregnancy sites might have also interfered with our experimental results and the sample size was relatively small. Third, our study utilized a single dose of LPS for intraperitoneal injection to induce a mouse model of PTB, lacking exploration of different doses and durations of LPS exposure, and the method of intraperitoneal LPS injection for inducing PTB is less commonly used compared to ultrasound-guided intrauterine or intra-amniotic LPS injection. Lastly, there are significant differences between human and mouse pregnancy tissues and labor processes, and we must be cautious when referencing these results to human studies.

In conclusion, this study presents a thorough investigation of differences in mRNAs and metabolites among various tissues in a mouse model of LPS-induced PTB. By integrating metabolomics and transcriptomics analyses, we have provided comprehensive evidence of the distinct molecular characteristics of the placenta, chorioamniotic membrane, decidua, uterus, and peripheral blood in this model. Our findings support the idea that PTB is an inflammatory syndrome, as most co-expressed DEGs and DEMs are linked to inflammation. A comprehensive analysis of these molecular changes could help identify common pathways and shed light on the underlying mechanisms of PTB initiation and labor in mice treated with intraperitoneal LPS. However, it is important to note that the specific role of these co-expressed DEGs and DEMs in triggering the inflammatory process associated with PTB or labor remains unclear, and further research is needed to elucidate their functions and potential clinical applications.

## Data availability statement

The datasets presented in this study can be found in online repositories. The names of the repository/repositories and accession number(s) can be found below: PRJNA917515 (SRA) and MTBLS6775 (Metabolights).

## Ethics statement

The animal study was approved by Ethics Committee of Fudan University (Protocol Number: 20211203S). The study was conducted in accordance with the local legislation and institutional requirements.

## Author contributions

XC is responsible for the experimental part and literature writing. XZ is responsible for auxiliary revision. SC and CX are responsible for reviewing the revised manuscript. All authors contributed to the article and approved the submitted version.

## Supplementary material

Supplementary Figure 1 LPS-induced PTB model in Mice by intraperitoneal injection. **(A)** The time from LPS or PBS injection to the birth of the first pup. **(B)** The ratio of fetal weight to placental weight at 12 h after LPS and PBS injection. **P* < 0.05, ***P* < 0.01.

Supplementary Figure 2 Two-dimensional principal component analysis (PCA) plot. **(A)** Placenta (LPS vs. PBS). **(B)** Chorioamniotic membrane (LPS vs. PBS). **(C)** Decidua (LPS vs. PBS). **(D)** Uterus (LPS vs. PBS). **(E)** Peripheral blood (LPS vs. PBS). The points of the same color in the image represent each biological repetition in the group, and the distance between the points represents the overall expression difference of the sample. PC1 and PC2 represent different principal components, and the number in brackets represents the principal component interpretation. The higher the degree of interpretation of the principal component, the greater the overall gene expression difference represented by the distance between sample points and points in the dimension of the principal component vector.

Supplementary Figure 3 Volcano plot for differential gene expression. **(A)** Placenta (LPS vs. PBS). **(B)** Chorioamniotic membrane (LPS vs. PBS). **(C)** Decidua (LPS vs. PBS). **(D)** Uterus (LPS vs. PBS). **(E)** Peripheral blood (LPS vs. PBS). The abscissa represents the fold change of gene expression in different samples, and the ordinate represents the statistical significance of gene expression changes. The scattered points in the figure represent each gene, the gray dots represent genes with no significant difference, the red dots represent significantly upregulated genes, and the blue dots represent significantly down-regulated genes.

Supplementary Figure 4 Positive and negative ion mode OPLS-DA permutation test. **(A)** Placenta (POS-LPS vs. PBS). **(B)** Placenta (NEG-LPS vs. PBS). **(C)** Uterus (POS-LPS vs. PBS). **(D)** Uterus (NEG-LPS vs. PBS). In the figure, the abscissa represents the replacement retention, that is, the proportion consistent with the order of the original model Y variables, and the ordinate represents the values of R^2^ and Q^2^. The green dot represents R^2^, the blue dot represents Q^2^, and the two dotted lines represent the tropic of R^2^ and Q^2^, respectively. The R^2^ and Q^2^ in the upper right corner indicate that the permutation retention is equal to 1, which is the R^2^ and Q^2^ values of the original model.

Supplementary Figure 5 GSEA enrichment analysis result of DEGs. **(A)** Placenta (LPS vs. PBS). **(B)** Chorioamniotic membrane (LPS vs. PBS). **(C)** Decidua (LPS vs. PBS). **(D)** Uterus (LPS vs. PBS). **(E)** Peripheral blood (LPS vs. PBS). The graph is divided into four parts, from top to bottom, as follows: ① The distribution curve of enrichment score (ES), with the green line representing the ES distribution of all genes. The curve corresponds to the enrichment score of the gene set at the position with the highest absolute value on the Y-axis. When ES > 0, the left side of the peak is the core gene, and ES < 0, the right side of the peak is the core gene; ② Gene distribution map of the gene set, with vertical lines indicating the position of the genes in the entire sequence; ③ The color bar represents the color mapping of the sorting matrix, with positive values corresponding to red and negative values corresponding to blue. The intensity of the color increases with the magnitude of the value, meaning that larger values appear redder and smaller values appear bluer. Values closer to 0 are represented as white. ④ Sort matrix distribution diagram. GSEA, gene set enrichment analysis.

Supplementary Figure 6 Pie chart of transcription factor analysis. **(A)** Placenta (LPS vs. PBS). **(B)** Chorioamniotic membrane (LPS vs. PBS). **(C)** Decidua (LPS vs. PBS). **(D)** Uterus (LPS vs. PBS). **(E)** Peripheral blood (LPS vs. PBS). The AnimalTFDB database was screened according to gene ID, and the DEGs were annotated with transcription factors.

Supplementary Figure 7 Alternative splicing events in different mouse tissues. **(A)** Placenta (LPS vs. PBS). **(B)** Chorioamniotic membrane (LPS vs. PBS). **(C)** Decidua (LPS vs. PBS). **(D)** Uterus (LPS vs. PBS). **(E)** Peripheral blood (LPS vs. PBS). SE, Skipped exon; MXE, Mutually exclusive exon; A5SS, Alternative 5’ splice site; A3SS, Alternative 3’ splice site; RI, Retained intron.

Supplementary Figure 8 KEGG pathway enrichment analysis of alternative splicing genes. **(A)** Placenta (LPS vs. PBS). **(B)** Chorioamniotic membrane (LPS vs. PBS). **(C)** Decidua (LPS vs. PBS). **(D)** Uterus (LPS vs. PBS). **(E)** Peripheral blood (LPS vs. PBS). In this diagram, the degree of KEGG enrichment was measured by the Rich factor, *P*-value and the number of genes enriched in a given pathway. Rich factor refers to the ratio of the number of DEGs in the pathway to the total number of genes. Bubble size represents the number of DEGs (*P* adj < 0.05, |log2(fold change)| > 1) involved in the KEGG enrichment. Each point represents a KEGG pathway, the ordinate represents the pathway name, and the abscissa represents the log10(*P* value). The Rich factor is represented by color, with a darker red color indicating a higher level of significance.

Supplementary Figures 9, S10
Hierarchical clustering thermogram of DEMs of positive and negative ion modes. **(A)** Placenta (POS-LPS vs. PBS). **(B)** Placenta (NEG-LPS vs. PBS). **(C)** Uterus (POS-LPS vs. PBS). **(D)** Uterus (NEG-LPS vs. PBS). The ordinate in the diagram represents the metabolites with significant differential expression, and the abscissa represents the sample information. The color blocks at different positions represent the relative expression levels of metabolites at the corresponding positions. Red represents a relatively high expression level, and blue represents a relatively low expression level. Metabolites with similar expression patterns are clustered under the same cluster on the left side.

Supplementary Figure 11 Bubble Maps of KEGG Pathway Enrichment of DEMs. **(A)** Placenta (LPS vs. PBS). **(B)** Uterus (LPS vs. PBS). In this diagram, the degree of KEGG enrichment was measured by the Rich factor, P-value and the number of genes enriched in a given pathway. Rich factor refers to the ratio of the number of DEGs in the pathway to the total number of genes. P value refers to the significance of pathway enrichment, and the value range is [0,1]. The closer it is to zero, the more significant the enrichment is. Bubble size represents the number of DEGs (P adj < 0.05, |log2(fold change)| > 1) involved in the KEGG enrichment. Each point represents a KEGG pathway, the ordinate represents the pathway name, and the abscissa represents the log10(P value). The significance of the Rich factor is represented by color, with a darker red color indicating a higher level of richness.

Supplementary Figure 12 Network diagram of DEMs in positive and negative ion mode. **(A)** Placenta (POS-LPS vs. PBS). **(B)** Placenta (NEG-LPS vs. PBS). **(C)** Uterus (POS-LPS vs. PBS). **(D)** Uterus (NEG-LPS vs. PBS). Top 30 hub DEMs (*P* adj < 0.05, |log2(fold change)| > 1) identified using CytoHubba. Each circle in the figure represents a metabolite, and the lines between the circles indicate interactions between metabolites. The color of a circle reflects the number of interactions that the corresponding metabolite has, with a darker color indicating a higher degree of connectivity.

Supplementary Figure 13 Hierarchical clustering heatmap of Spearman correlation analysis of DEGs AND DEMs. **(A)** Placenta (LPS vs. PBS). **(B)** Uterus (LPS vs. PBS). In the hierarchical clustering heat map, each row represents a DEM, and each column represents a DEG. The left branch represents the difference in pairs. The upper branch represents the result of cluster analysis of DEGs. Each lattice in the hierarchical clustering heatmap contains two kinds of information (correlation coefficient r and *P*-value). The correlation coefficient r is represented by color, r> 0 represents a positive correlation, and r < 0 represents a negative correlation. A deeper color indicates a stronger correlation.

Supplementary Figure 14 Histogram of common KEGG enrichment pathway of DEGs and DEMs. **(A)** Placenta (LPS vs. PBS). **(B)** Uterus (LPS vs. PBS). Each column in the figure represents a KEGG pathway, with different colors representing different omics, among which blue represents transcriptome and orange represents metabolomics. The ordinate represents the name of the pathway, and the abscissa represents the significance of pathway enrichment. Columns with greater height indicate a higher level of enrichment of biological pathways in the measured samples and a more obvious change in activity.

Supplementary Figure 15 KEGG pathway annotation map of DEGs and DEMs. **(A, D, E)** Uterus (LPS vs. PBS). **(B, C)** Placenta (LPS vs. PBS). The box in the figure represents the gene product, and the circle represents the metabolite. The red or green background represents DEGs or DEMs.

Supplementary Figure 16 Correlation analysis network diagram of representative DEGs and DEMs. **(A)** Placenta (LPS vs. PBS). **(B)** Uterus (LPS vs. PBS). In the figure, red circles represent DEGs or DEMs (*P* adj < 0.05, |log2(fold change)| > 1), and the lines represent the correlation between the two.

Supplementary Table 1 Sample data quality summary.

Supplementary Table 2 Differentially expressed genes.

Supplementary Table 3 Co-expressed DEGs in different tissues.

Supplementary Table 4 Differential metabolites in the placenta and uterus under positive and negative ion mode.

Supplementary Table 5 GO, KEGG and TF target analysis table of DEGs in different tissues.

Supplementary Table 6 KEGG pathway enrichment analysis of common DEGs.

Supplementary Table 7 Placental and uterine differential genes and differential metabolites correlation statistics table.
