# Supplementary figures and images for: Integration of transcriptomics and metabolomics reveals the responses of the maternal circulation and maternal-fetal interface to LPS-induced preterm birth in mice
Source: Front Immunol. 2023 Aug 15;14:1213902. doi: 10.3389/fimmu.2023.1213902 (PMC10464907; doi:10.3389/fimmu.2023.1213902)

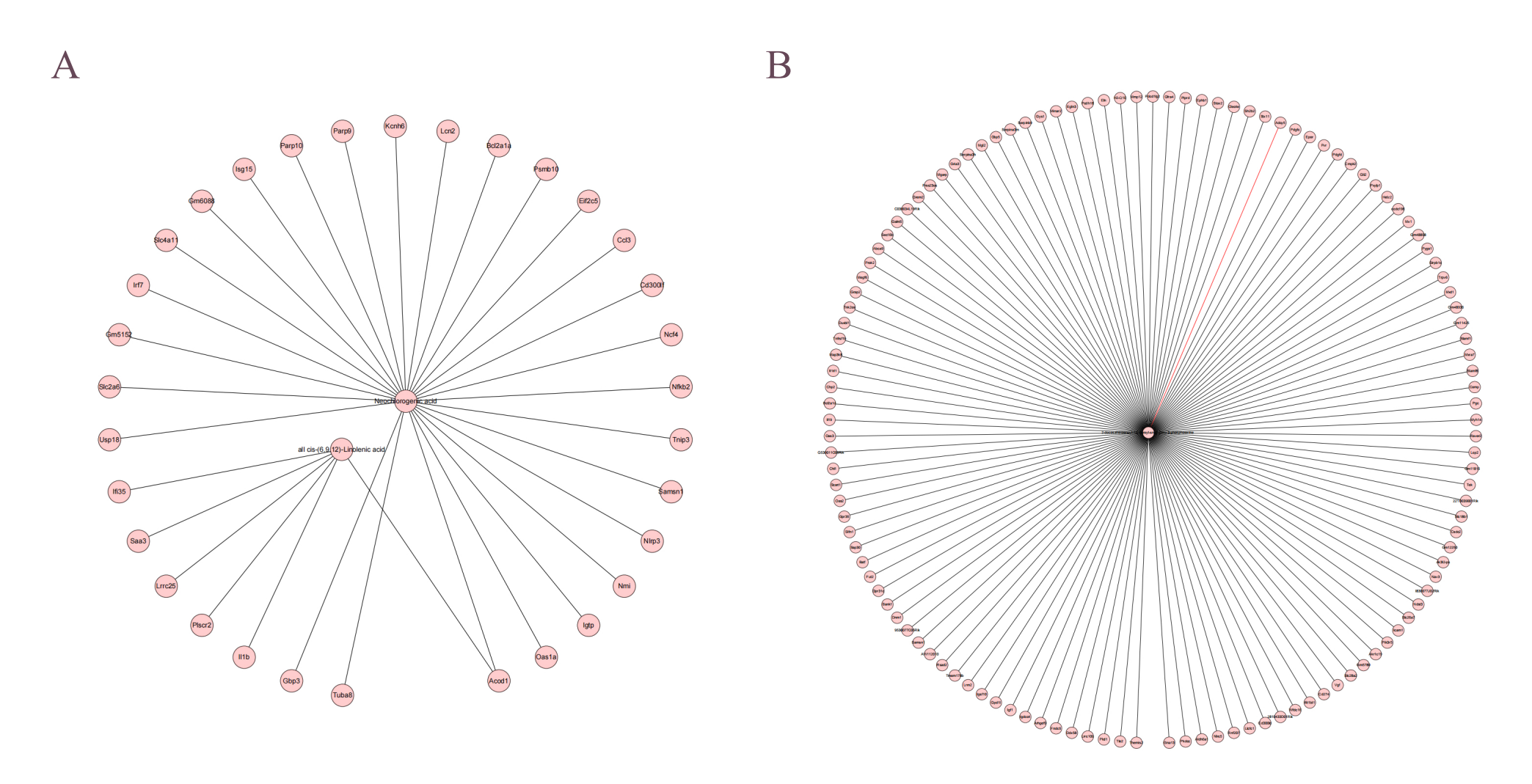

Supplement: Supplementary Figure 1 — LPS-induced PTB model in Mice by intraperitoneal injection. (A) The time from LPS or PBS injection to the birth of the first pup. (B) The ratio of fetal weight to placental weight at 12 h after LPS and PBS injection. *P < 0.05, **P < 0.01. [file Image_1.tif]

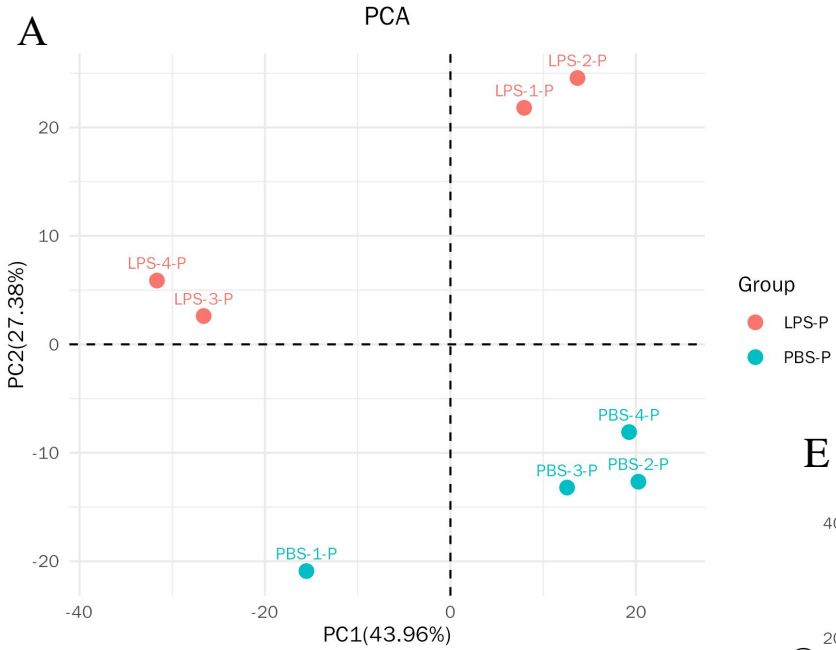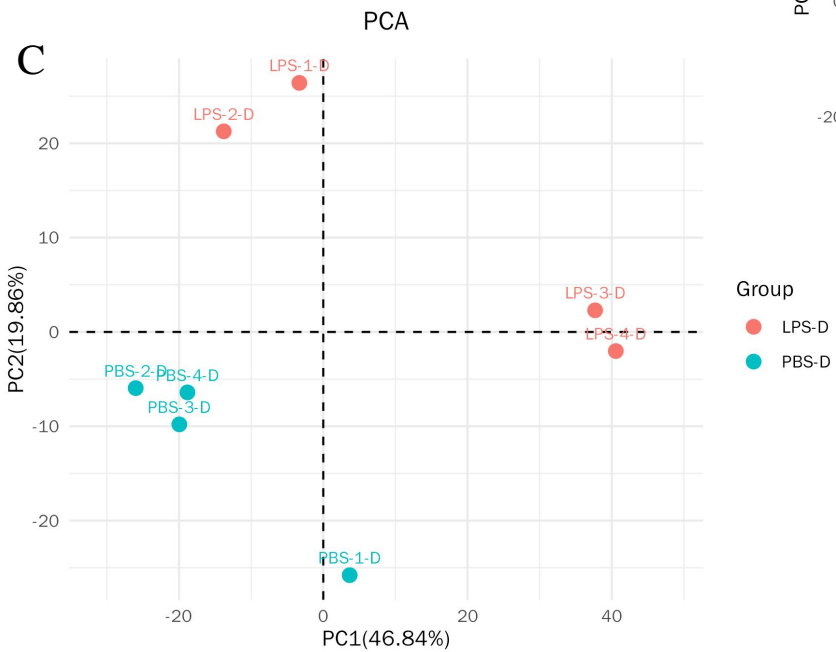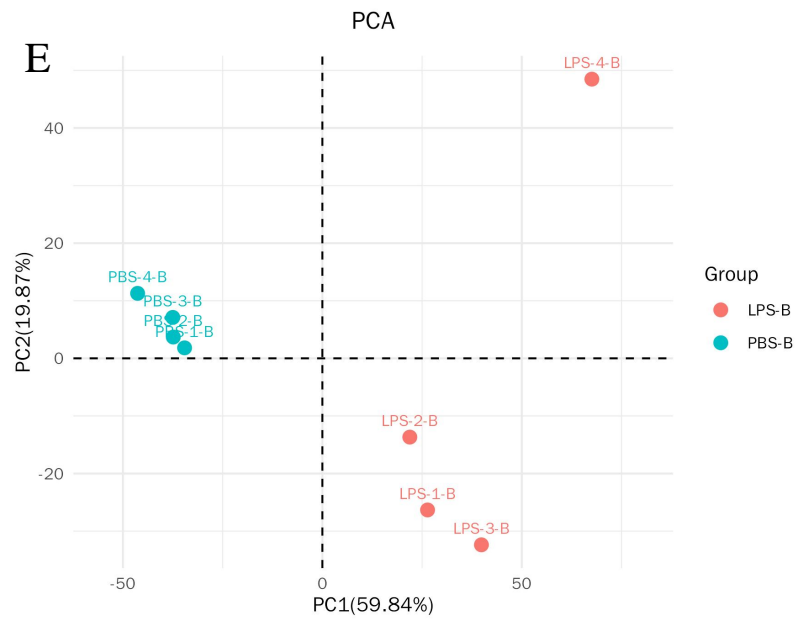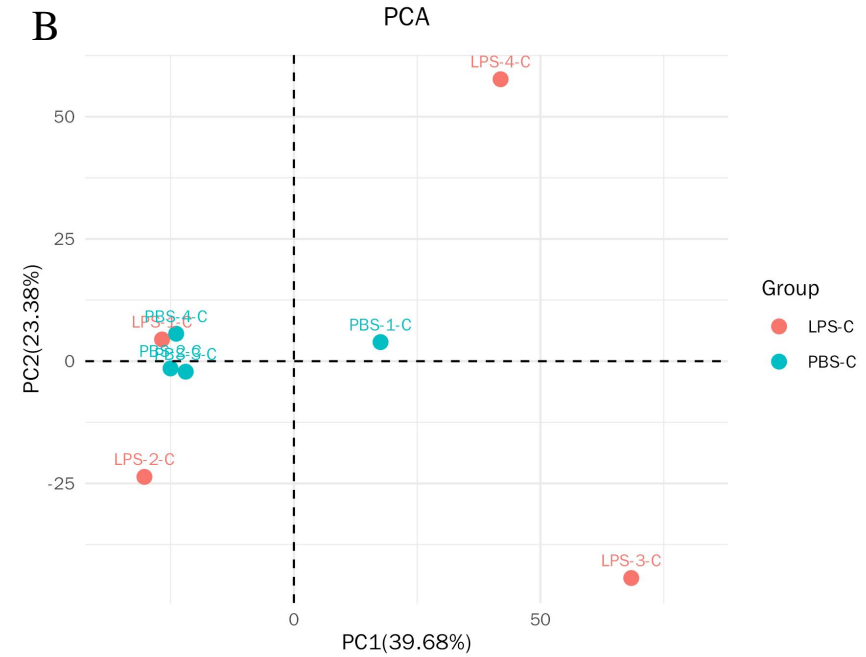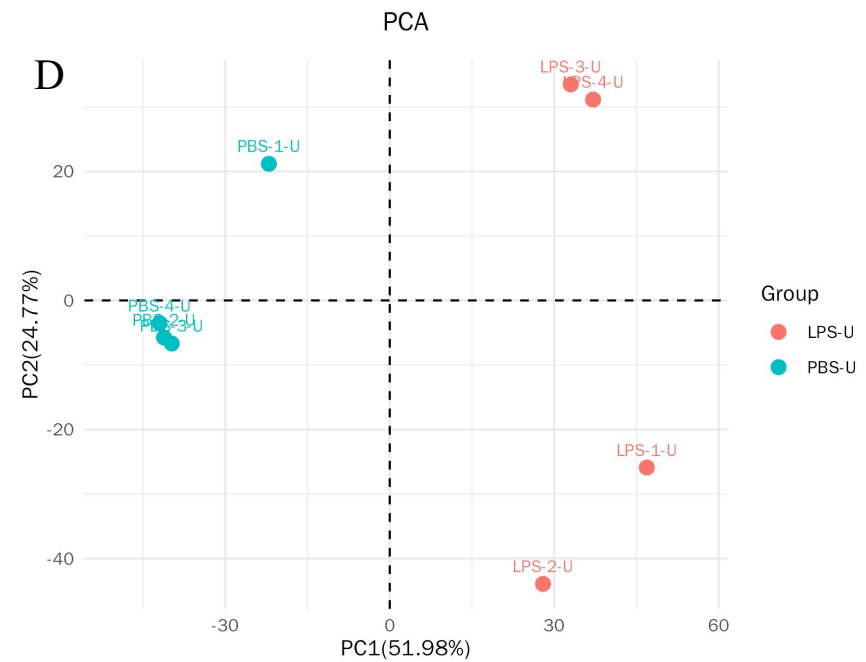

Supplement: Supplementary Figure 2 — Two-dimensional principal component analysis (PCA) plot. (A) Placenta (LPS vs. PBS). (B) Chorioamniotic membrane (LPS vs. PBS). (C) Decidua (LPS vs. PBS). (D) Uterus (LPS vs. PBS). (E) Peripheral blood (LPS vs. PBS). The points of the same color in the image represent each biological repetition in the group, and the distance between the points represents the overall expression difference of the sample. PC1 and PC2 represent different principal components, and the number in brackets represents the principal component interpretation. The higher the degree of interpretation of the principal component, the greater the overall gene expression difference represented by the distance between sample points and points in the dimension of the principal component vector. [file DataSheet_1.pdf]

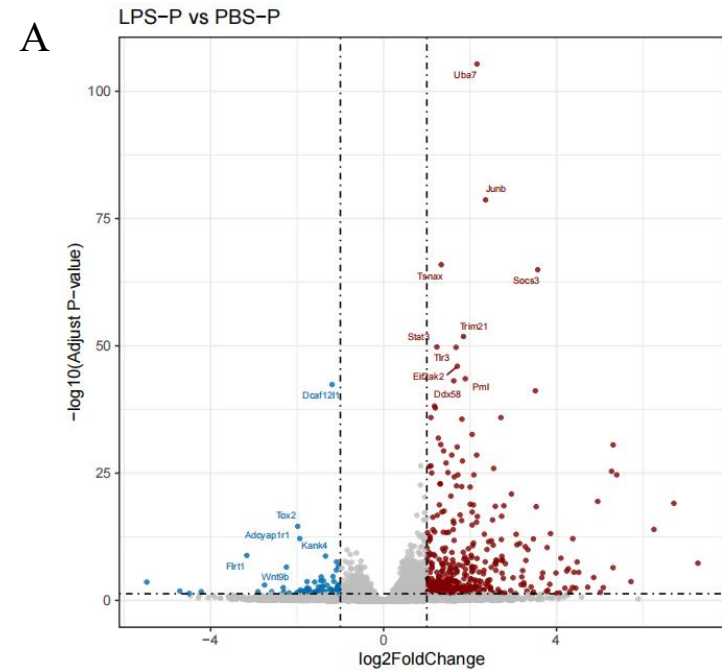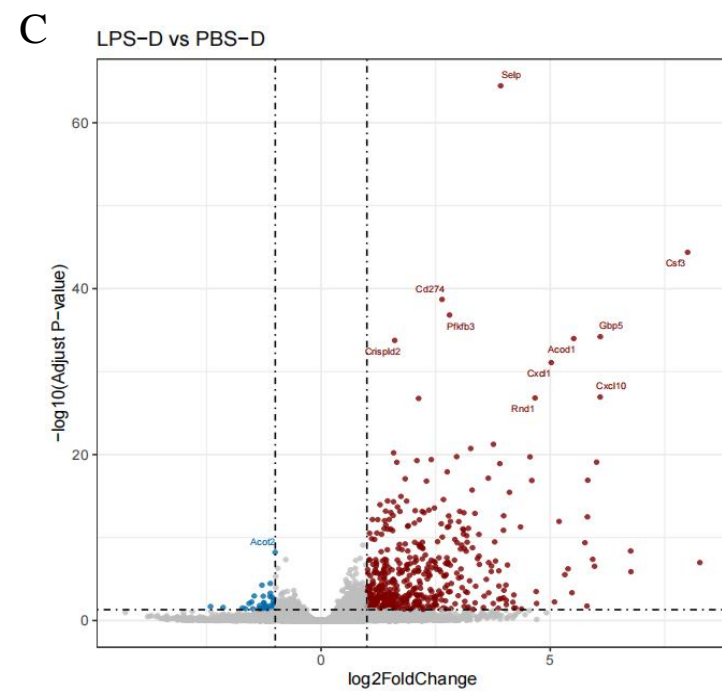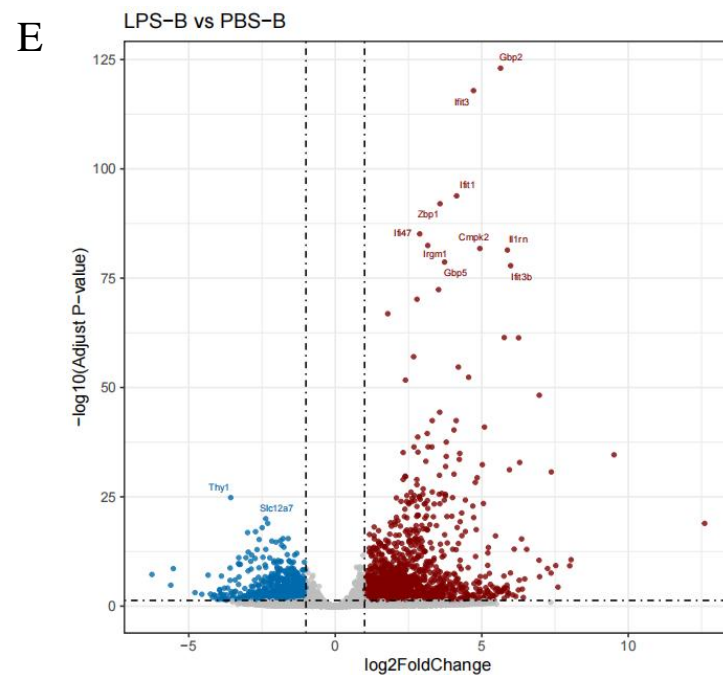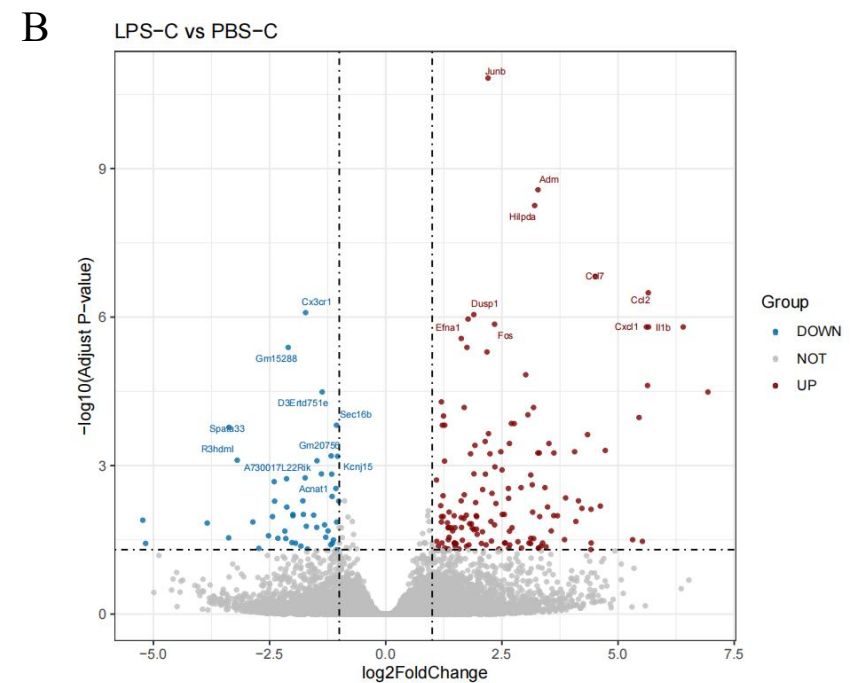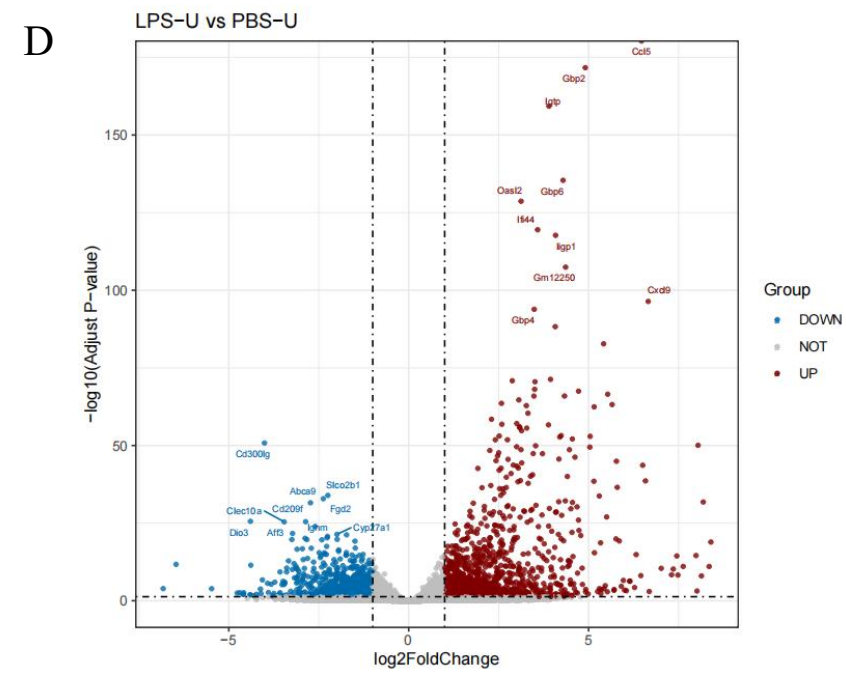

Supplement: Supplementary Figure 3 — Volcano plot for differential gene expression. (A) Placenta (LPS vs. PBS). (B) Chorioamniotic membrane (LPS vs. PBS). (C) Decidua (LPS vs. PBS). (D) Uterus (LPS vs. PBS). (E) Peripheral blood (LPS vs. PBS). The abscissa represents the fold change of gene expression in different samples, and the ordinate represents the statistical significance of gene expression changes. The scattered points in the figure represent each gene, the gray dots represent genes with no significant difference, the red dots represent significantly upregulated genes, and the blue dots represent significantly down-regulated genes. [file DataSheet_2.pdf]

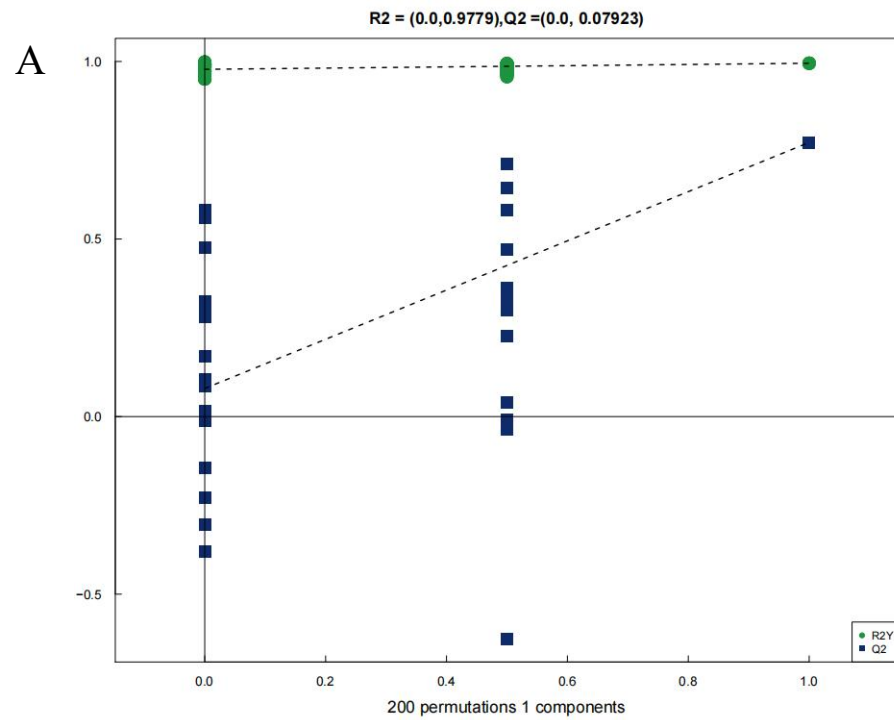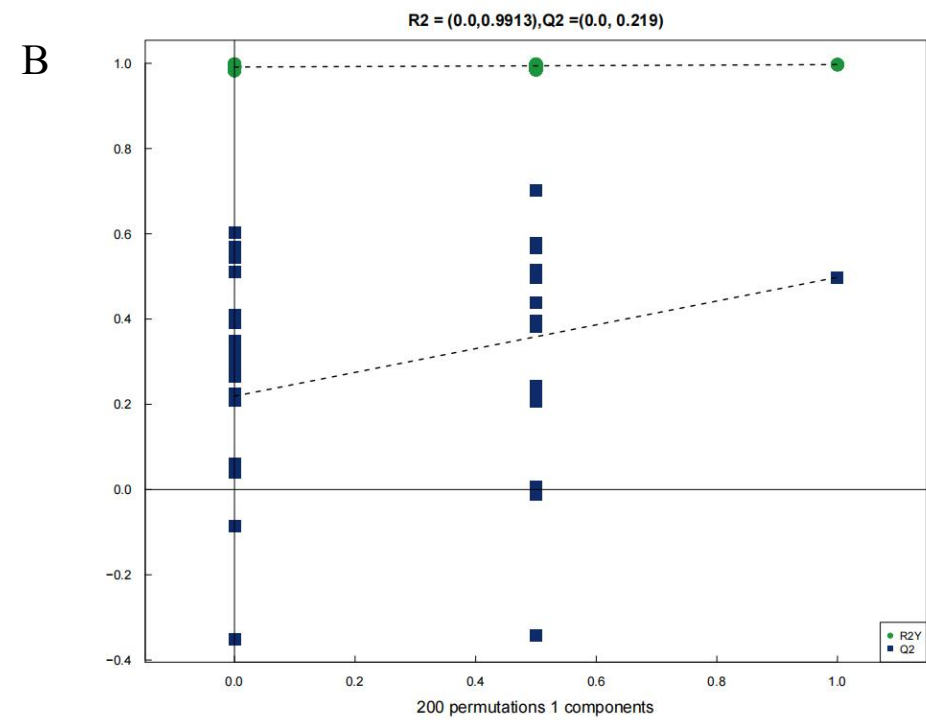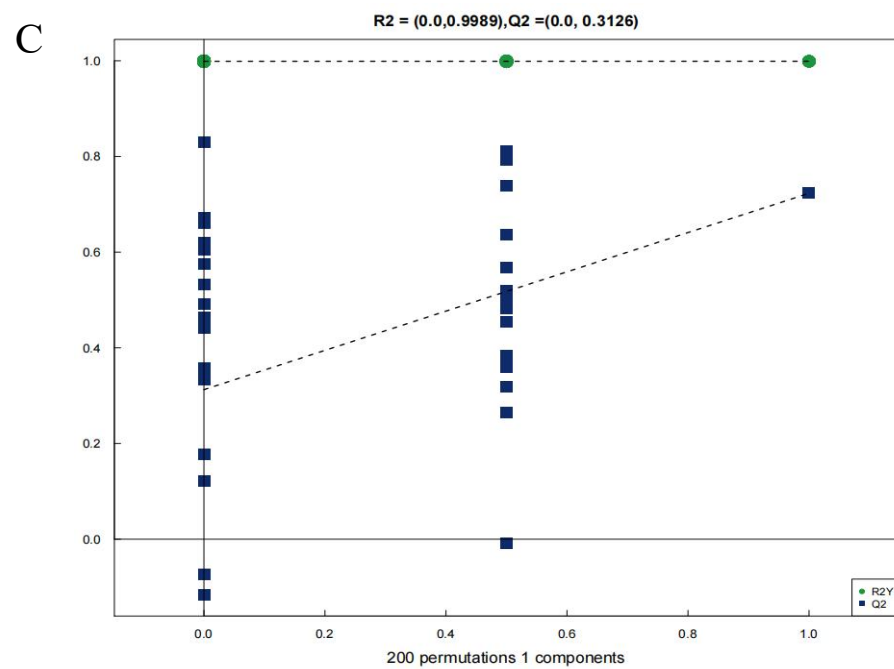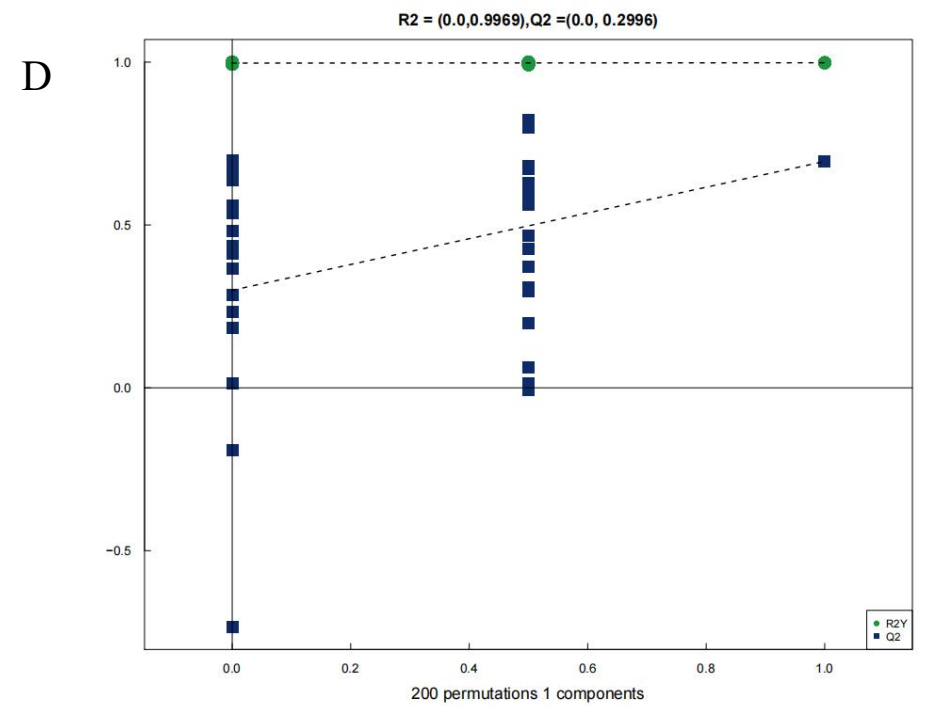

Supplement: Supplementary Figure 4 — Positive and negative ion mode OPLS-DA permutation test. (A) Placenta (POS-LPS vs. PBS). (B) Placenta (NEG-LPS vs. PBS). (C) Uterus (POS-LPS vs. PBS). (D) Uterus (NEG-LPS vs. PBS). In the figure, the abscissa represents the replacement retention, that is, the proportion consistent with the order of the original model Y variables, and the ordinate represents the values of R2 and Q2. The green dot represents R2, the blue dot represents Q2, and the two dotted lines represent the tropic of R2 and Q2, respectively. The R2 and Q2 in the upper right corner indicate that the permutation retention is equal to 1, which is the R2 and Q2 values of the original model. [file DataSheet_3.pdf]

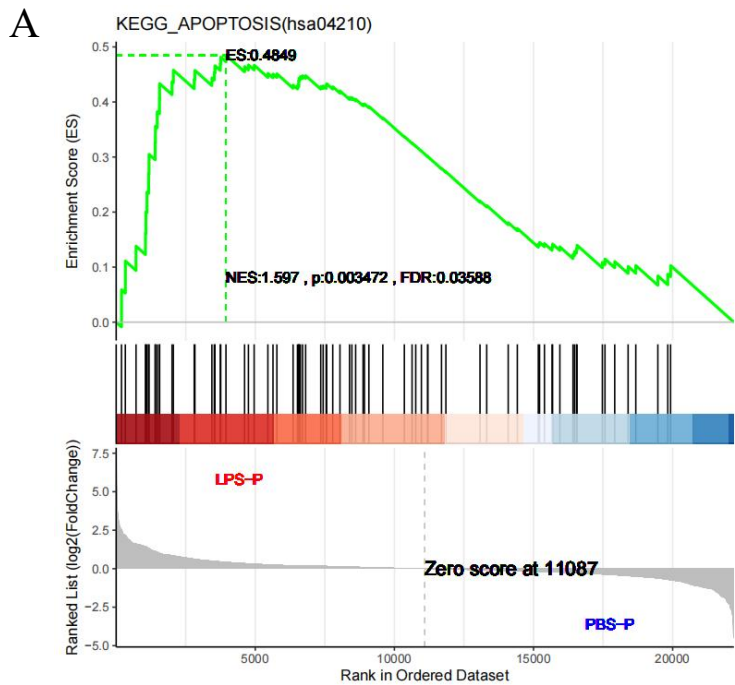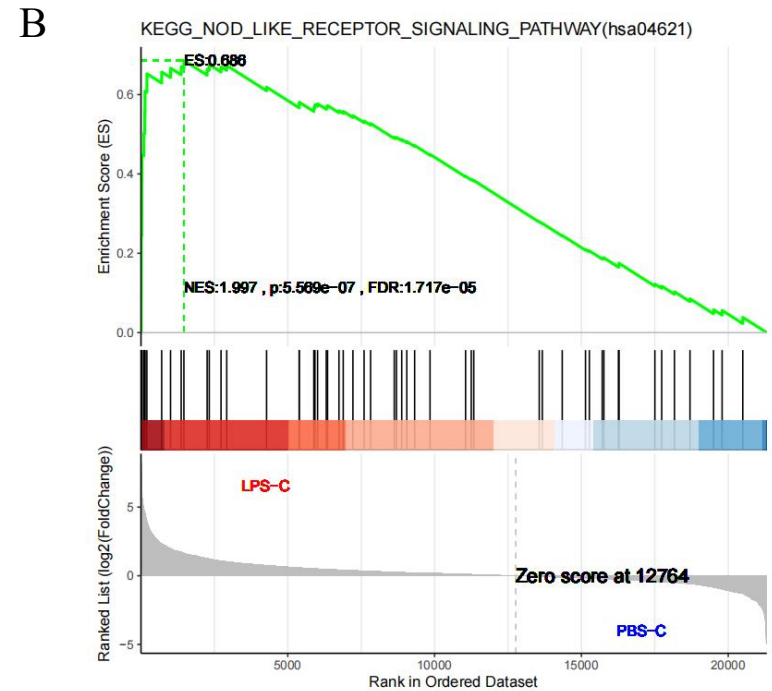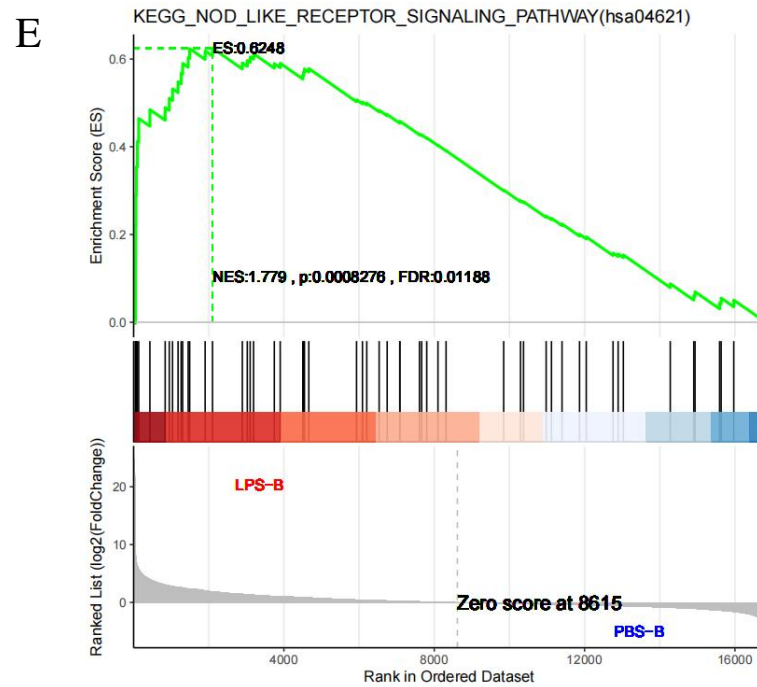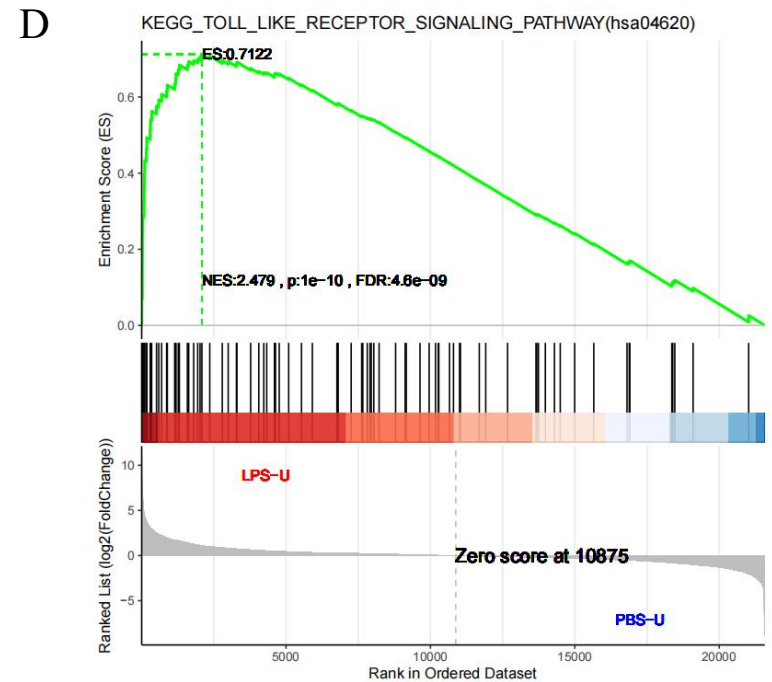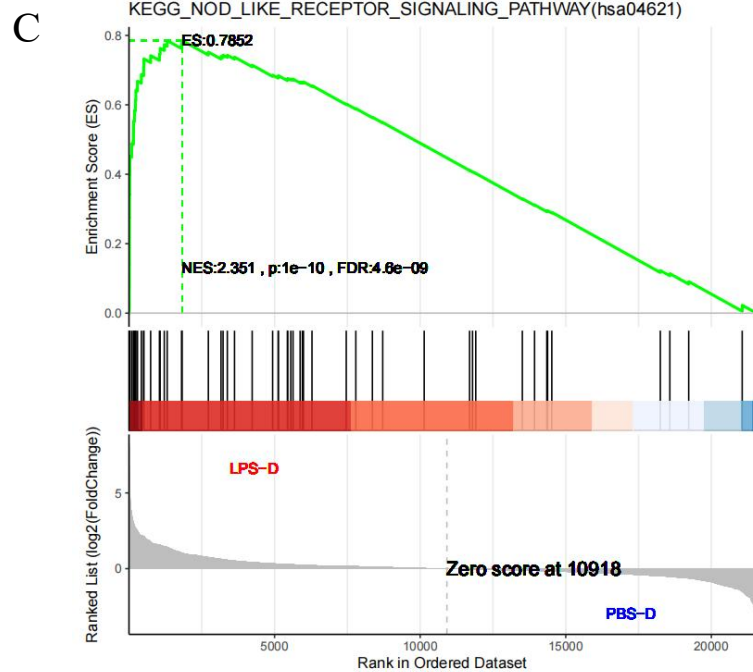

Supplement: Supplementary Figure 5 — GSEA enrichment analysis result of DEGs. (A) Placenta (LPS vs. PBS). (B) Chorioamniotic membrane (LPS vs. PBS). (C) Decidua (LPS vs. PBS). (D) Uterus (LPS vs. PBS). (E) Peripheral blood (LPS vs. PBS). The graph is divided into four parts, from top to bottom, as follows: ① The distribution curve of enrichment score (ES), with the green line representing the ES distribution of all genes. The curve corresponds to the enrichment score of the gene set at the position with the highest absolute value on the Y-axis. When ES > 0, the left side of the peak is the core gene, and ES < 0, the right side of the peak is the core gene; ② Gene distribution map of the gene set, with vertical lines indicating the position of the genes in the entire sequence; ③ The color bar represents the color mapping of the sorting matrix, with positive values corresponding to red and negative values corresponding to blue. The intensity of the color increases with the magnitude of the value, meaning that larger values appear redder and smaller values appear bluer. Values closer to 0 are represented as white. ④ Sort matrix distribution diagram. GSEA, gene set enrichment analysis. [file DataSheet_4.pdf]

A

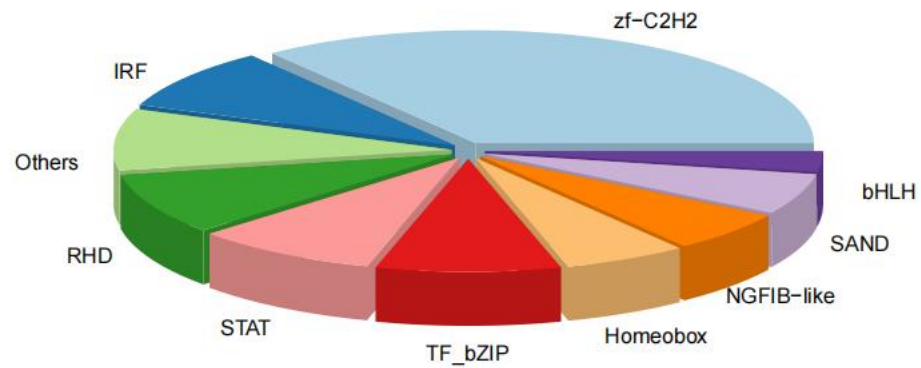

B

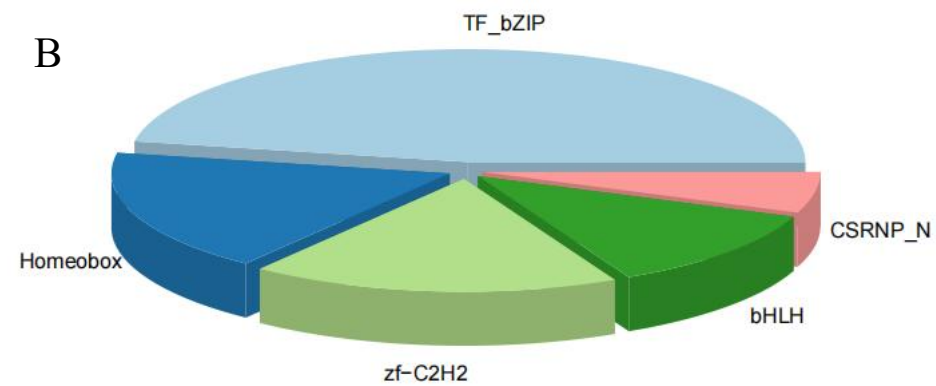

E

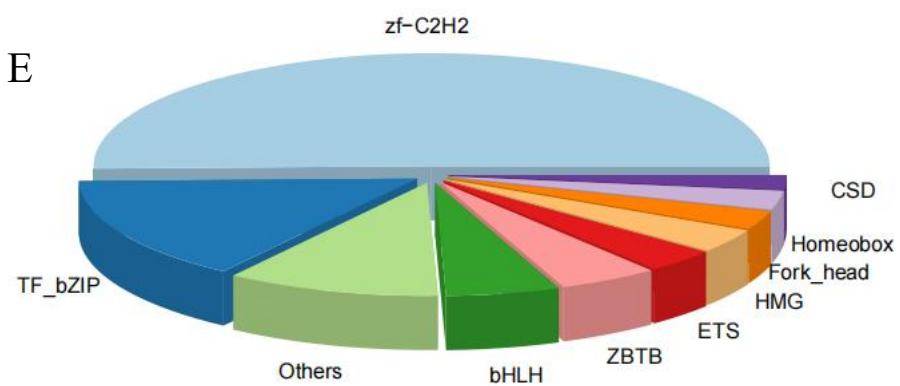

C

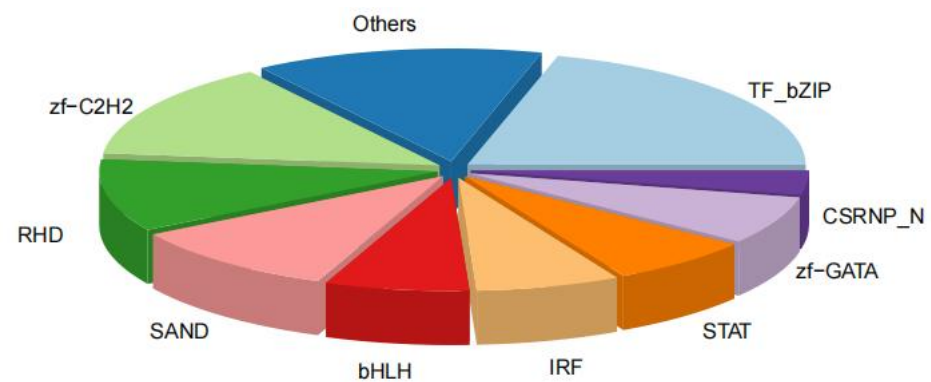

D

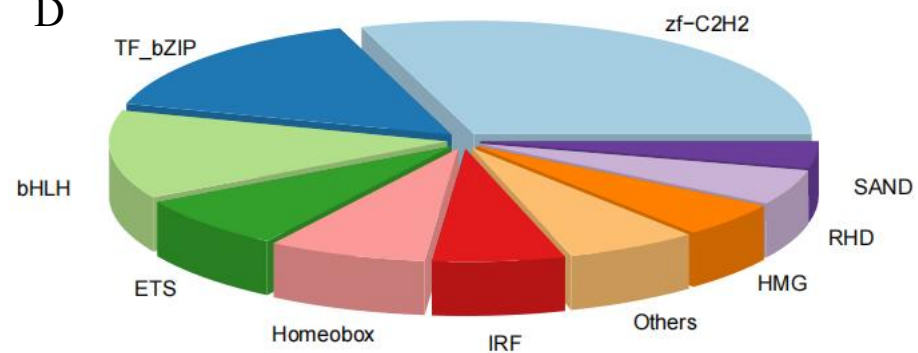

Supplement: Supplementary Figure 6 — Pie chart of transcription factor analysis. (A) Placenta (LPS vs. PBS). (B) Chorioamniotic membrane (LPS vs. PBS). (C) Decidua (LPS vs. PBS). (D) Uterus (LPS vs. PBS). (E) Peripheral blood (LPS vs. PBS). The AnimalTFDB database was screened according to gene ID, and the DEGs were annotated with transcription factors. [file DataSheet_5.pdf]

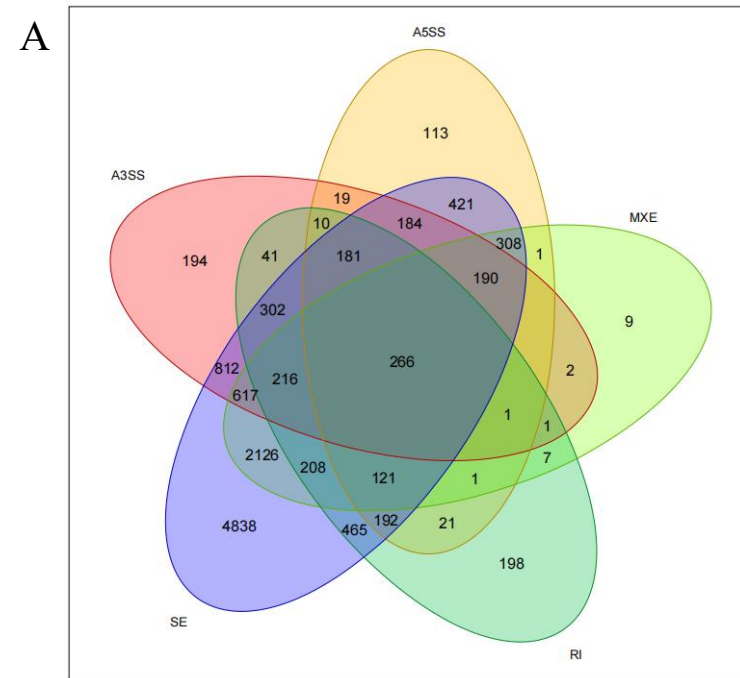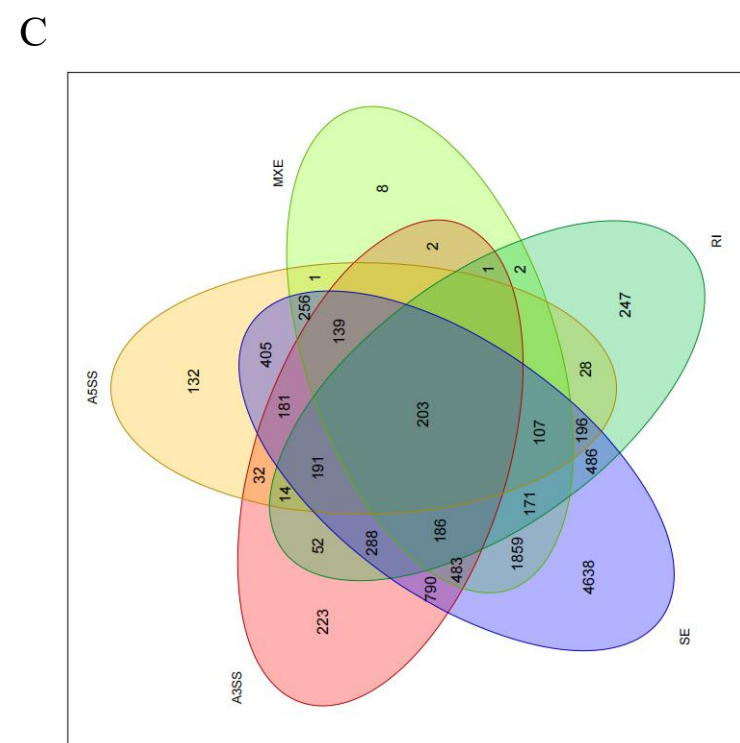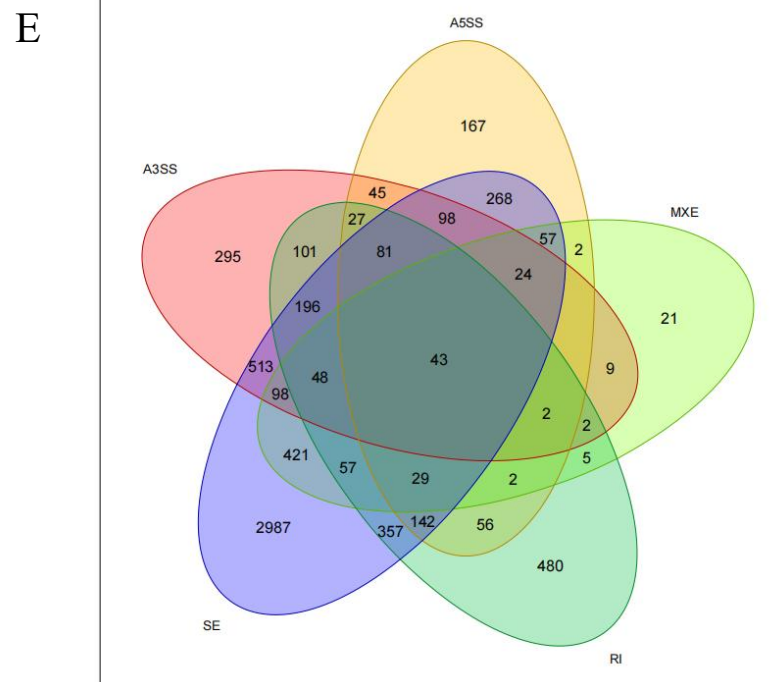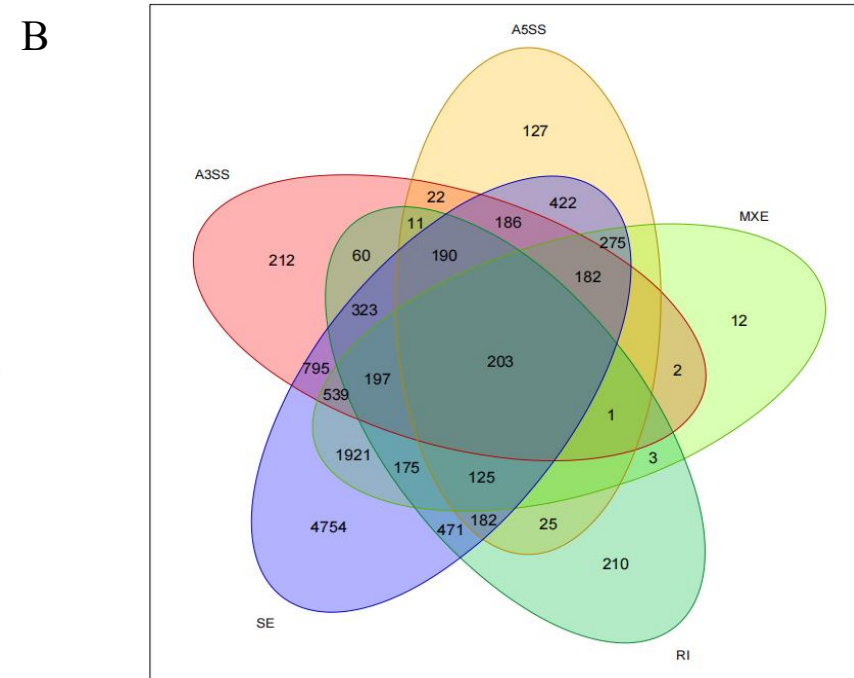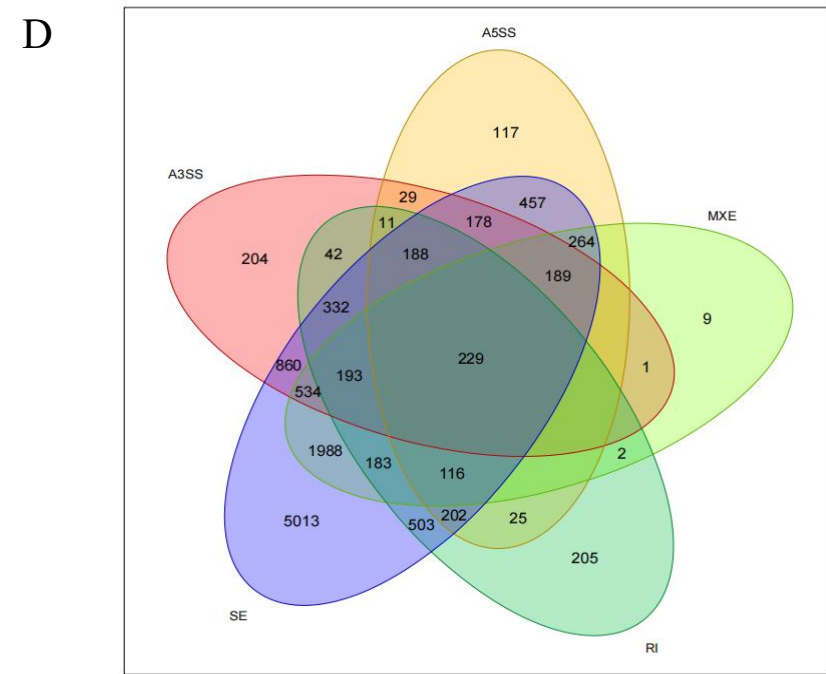

Supplement: Supplementary Figure 7 — Alternative splicing events in different mouse tissues. (A) Placenta (LPS vs. PBS). (B) Chorioamniotic membrane (LPS vs. PBS). (C) Decidua (LPS vs. PBS). (D) Uterus (LPS vs. PBS). (E) Peripheral blood (LPS vs. PBS). SE, Skipped exon; MXE, Mutually exclusive exon; A5SS, Alternative 5’ splice site; A3SS, Alternative 3’ splice site; RI, Retained intron. [file DataSheet_6.pdf]

A

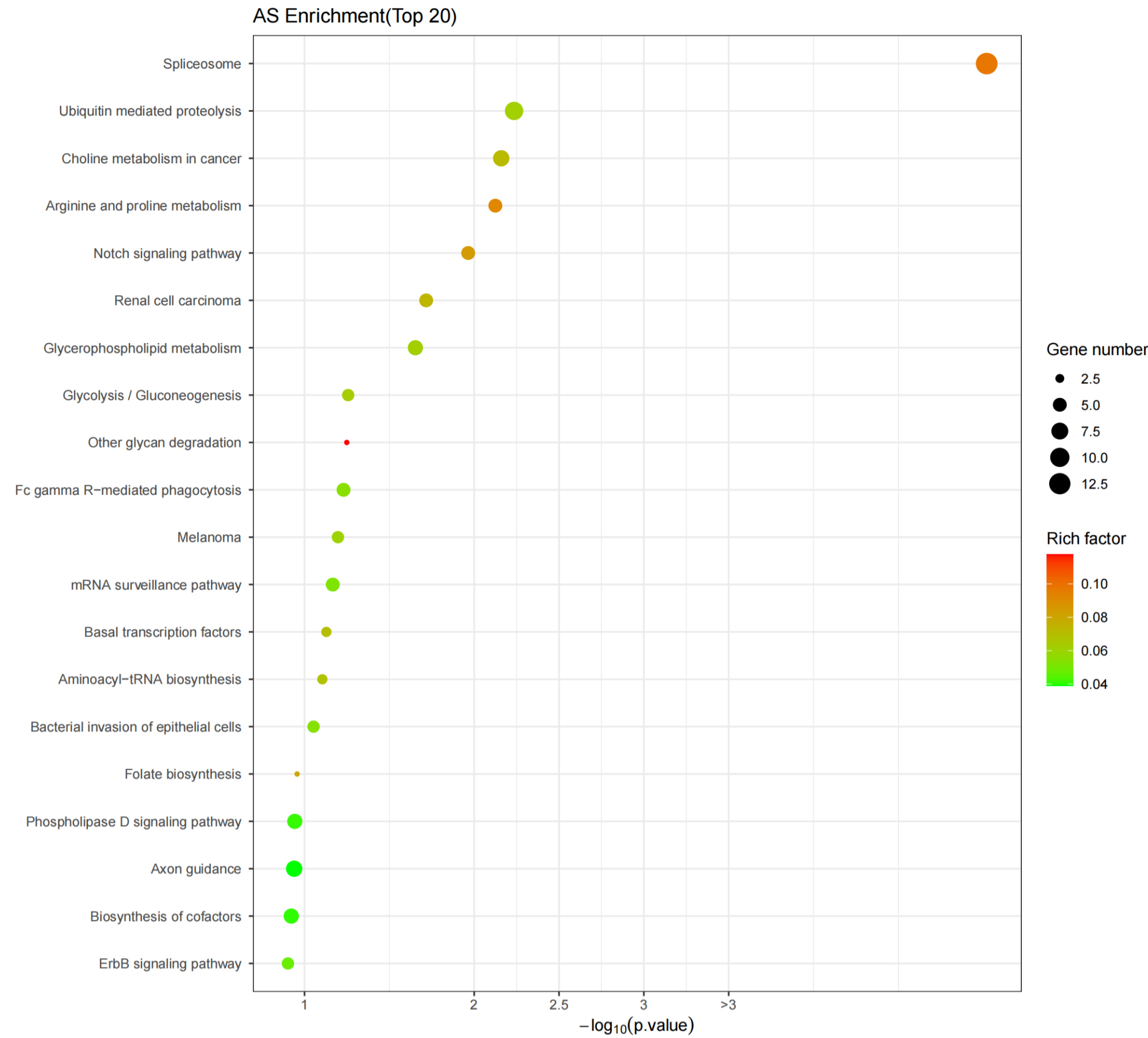

B

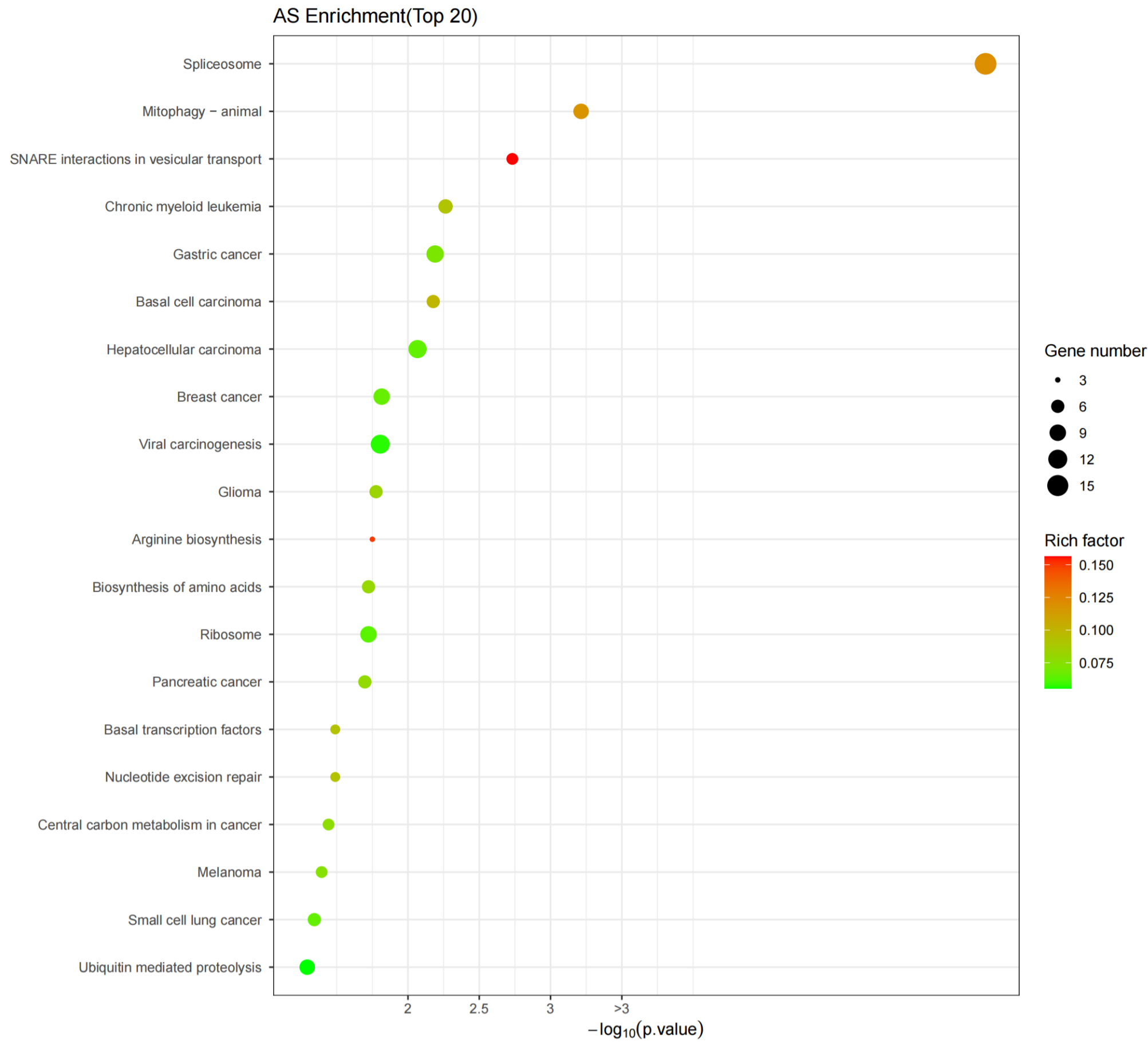

C

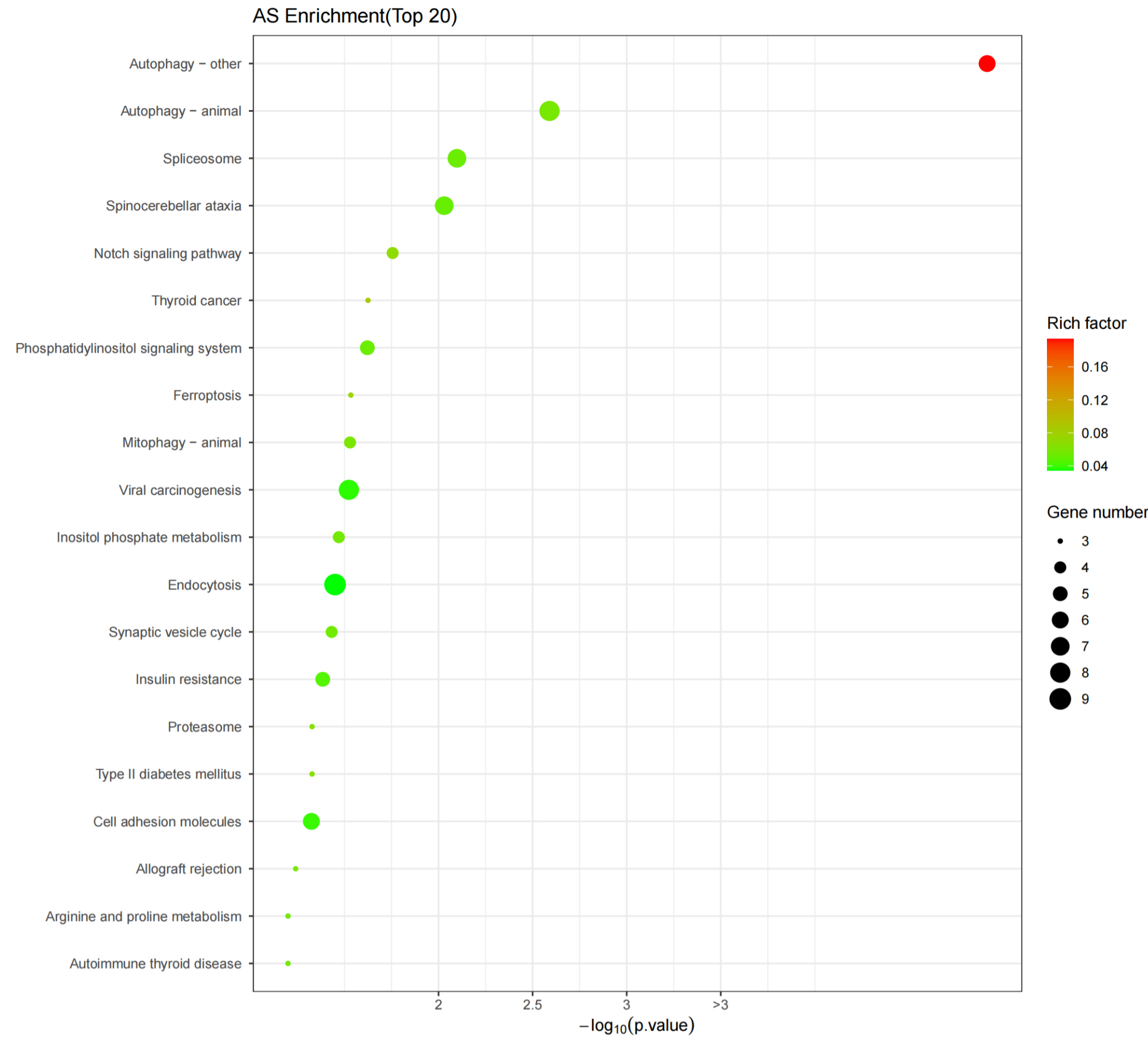

D

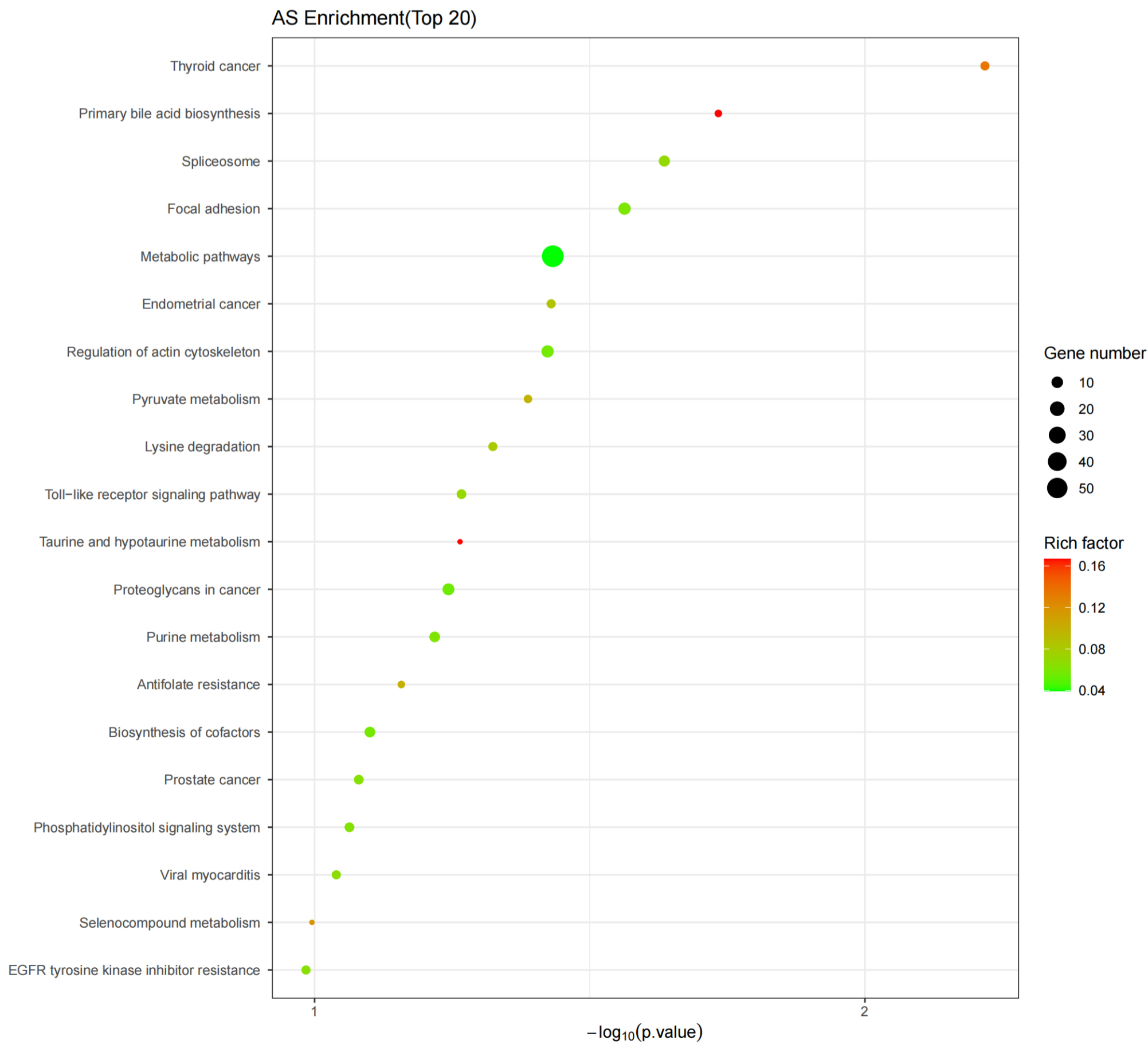

E

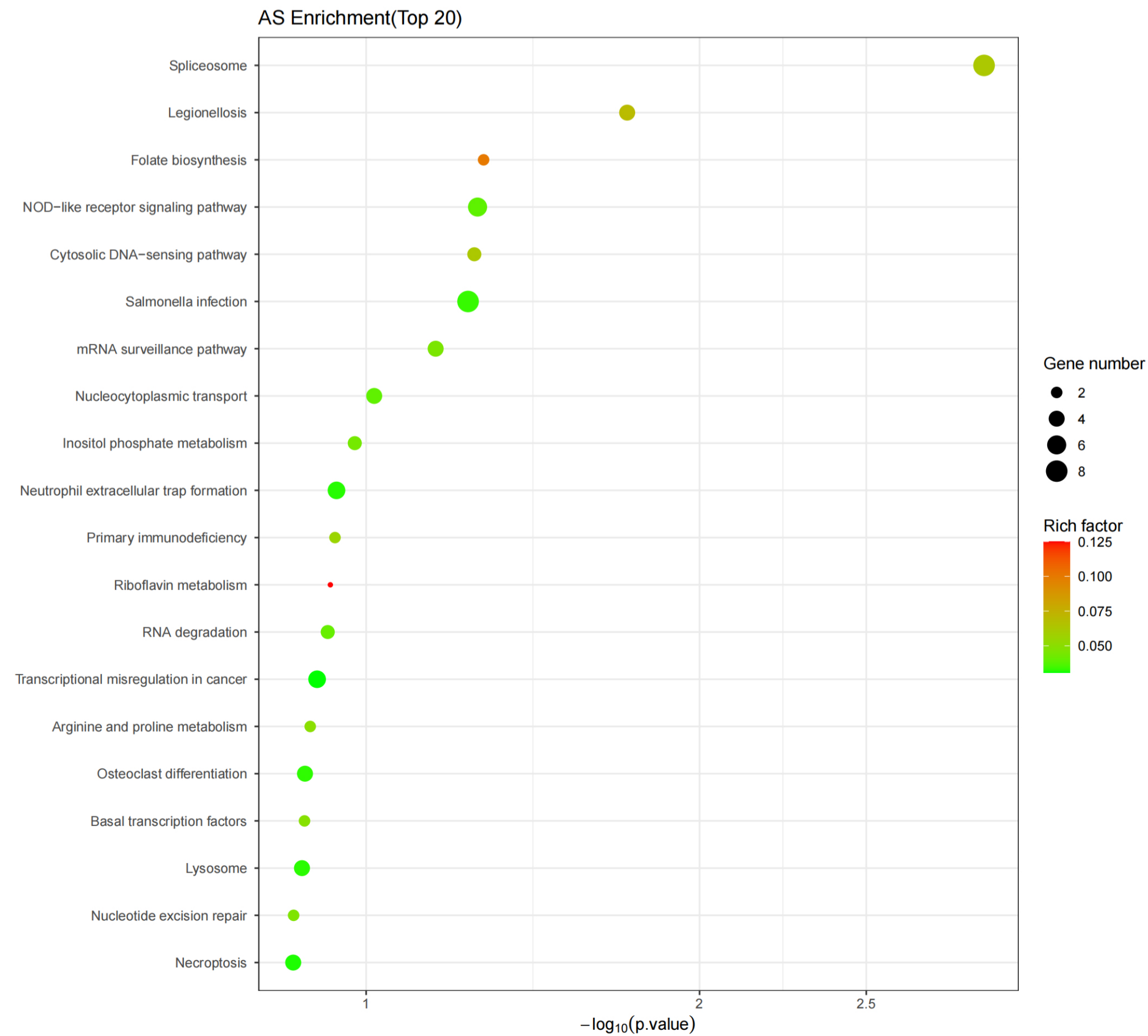

Supplement: Supplementary Figure 8 — KEGG pathway enrichment analysis of alternative splicing genes. (A) Placenta (LPS vs. PBS). (B) Chorioamniotic membrane (LPS vs. PBS). (C) Decidua (LPS vs. PBS). (D) Uterus (LPS vs. PBS). (E) Peripheral blood (LPS vs. PBS). In this diagram, the degree of KEGG enrichment was measured by the Rich factor, P-value and the number of genes enriched in a given pathway. Rich factor refers to the ratio of the number of DEGs in the pathway to the total number of genes. Bubble size represents the number of DEGs (P adj < 0.05, |log2(fold change)| > 1) involved in the KEGG enrichment. Each point represents a KEGG pathway, the ordinate represents the pathway name, and the abscissa represents the log10(P value). The Rich factor is represented by color, with a darker red color indicating a higher level of significance. [file DataSheet_7.pdf]

A

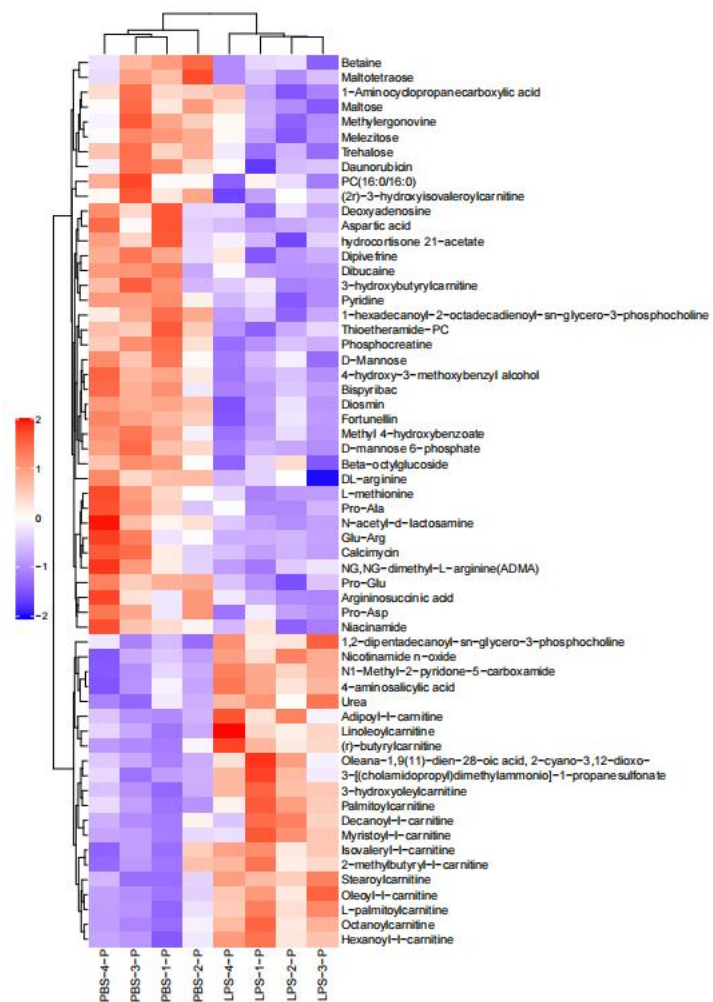

POS

B

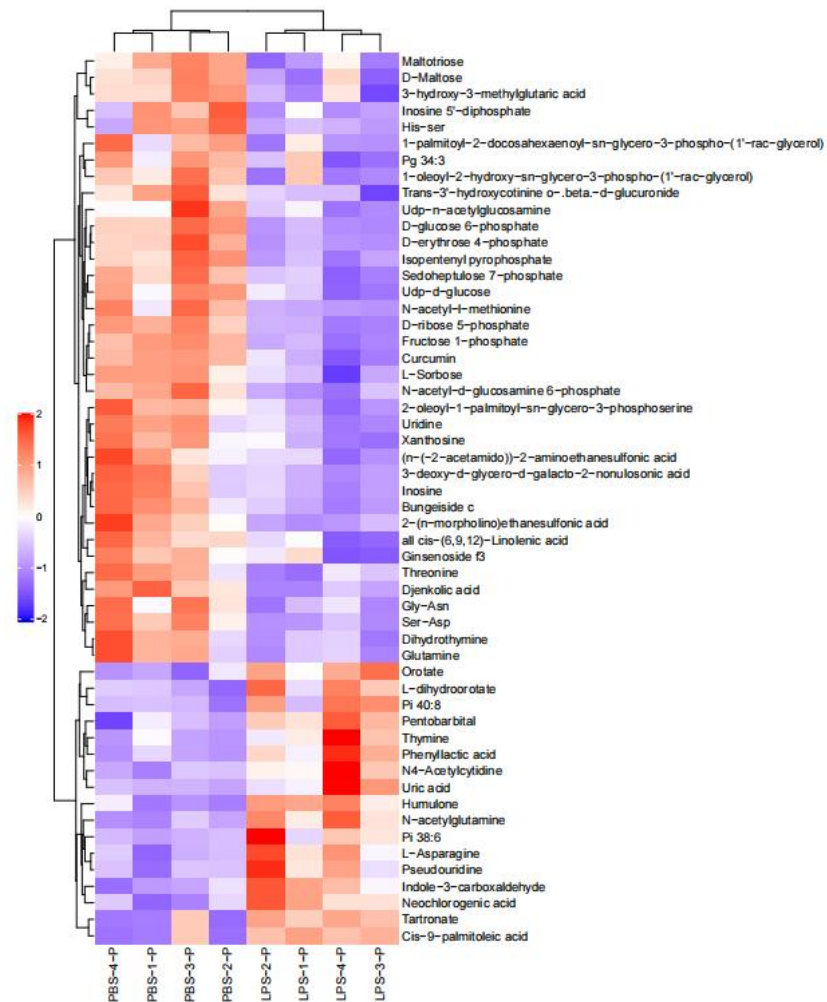

NEG

Supplement: Supplementary Figures 9, S10 — Hierarchical clustering thermogram of DEMs of positive and negative ion modes. (A) Placenta (POS-LPS vs. PBS). (B) Placenta (NEG-LPS vs. PBS). (C) Uterus (POS-LPS vs. PBS). (D) Uterus (NEG-LPS vs. PBS). The ordinate in the diagram represents the metabolites with significant differential expression, and the abscissa represents the sample information. The color blocks at different positions represent the relative expression levels of metabolites at the corresponding positions. Red represents a relatively high expression level, and blue represents a relatively low expression level. Metabolites with similar expression patterns are clustered under the same cluster on the left side. [file DataSheet_8.pdf]

C

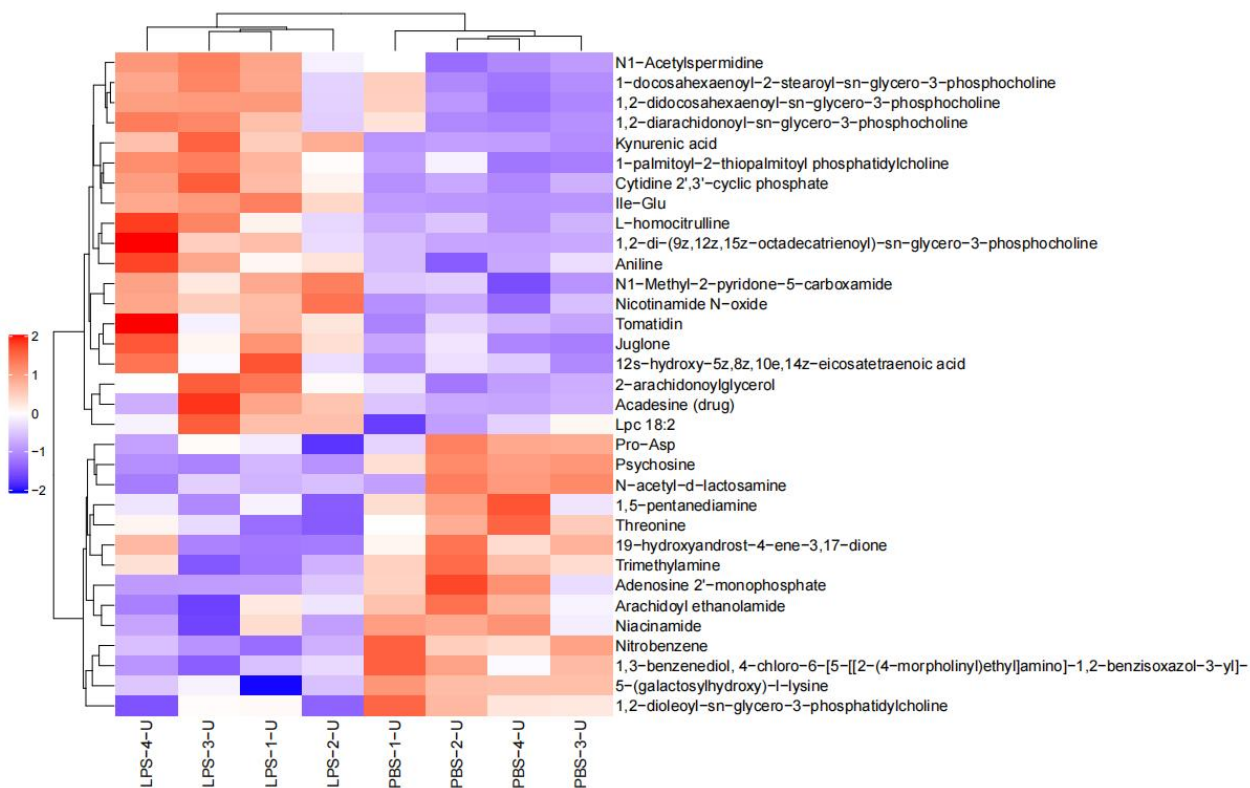

POS

D

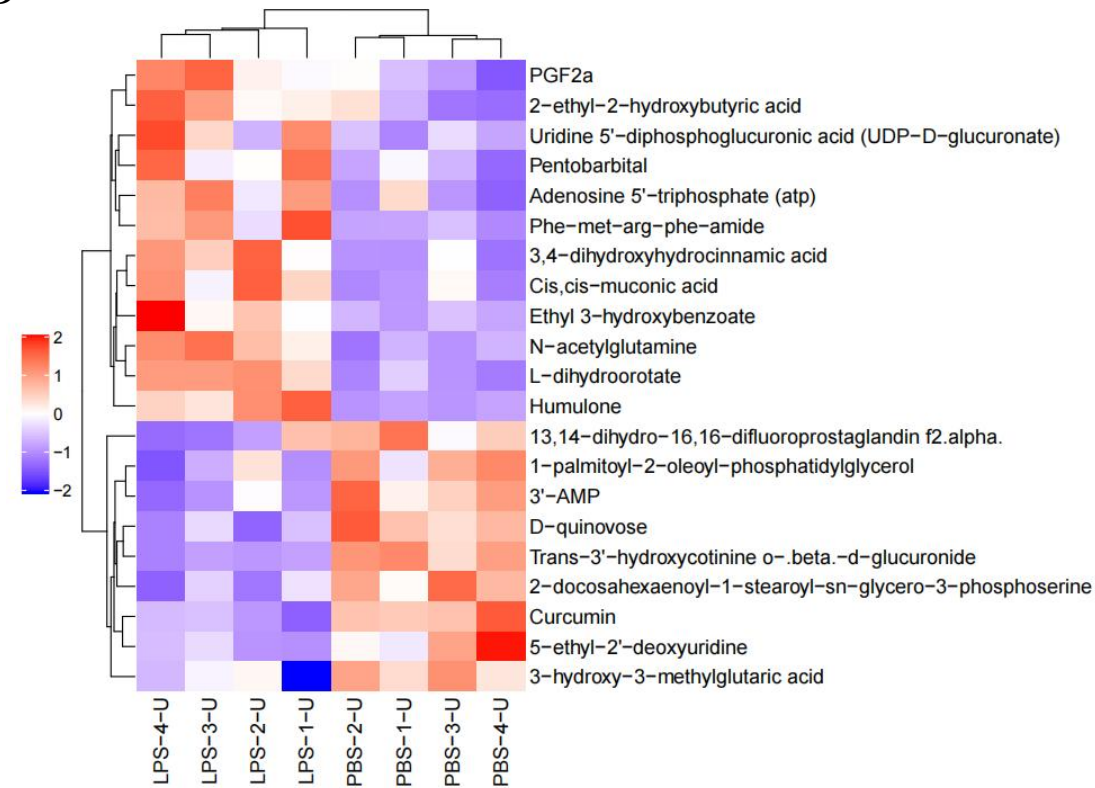

NEG

Supplement: Supplementary Figure 11 — Bubble Maps of KEGG Pathway Enrichment of DEMs. (A) Placenta (LPS vs. PBS). (B) Uterus (LPS vs. PBS). In this diagram, the degree of KEGG enrichment was measured by the Rich factor, P-value and the number of genes enriched in a given pathway. Rich factor refers to the ratio of the number of DEGs in the pathway to the total number of genes. P value refers to the significance of pathway enrichment, and the value range is [0,1]. The closer it is to zero, the more significant the enrichment is. Bubble size represents the number of DEGs (P adj < 0.05, |log2(fold change)| > 1) involved in the KEGG enrichment. Each point represents a KEGG pathway, the ordinate represents the pathway name, and the abscissa represents the log10(P value). The significance of the Rich factor is represented by color, with a darker red color indicating a higher level of richness. [file DataSheet_9.pdf]

A

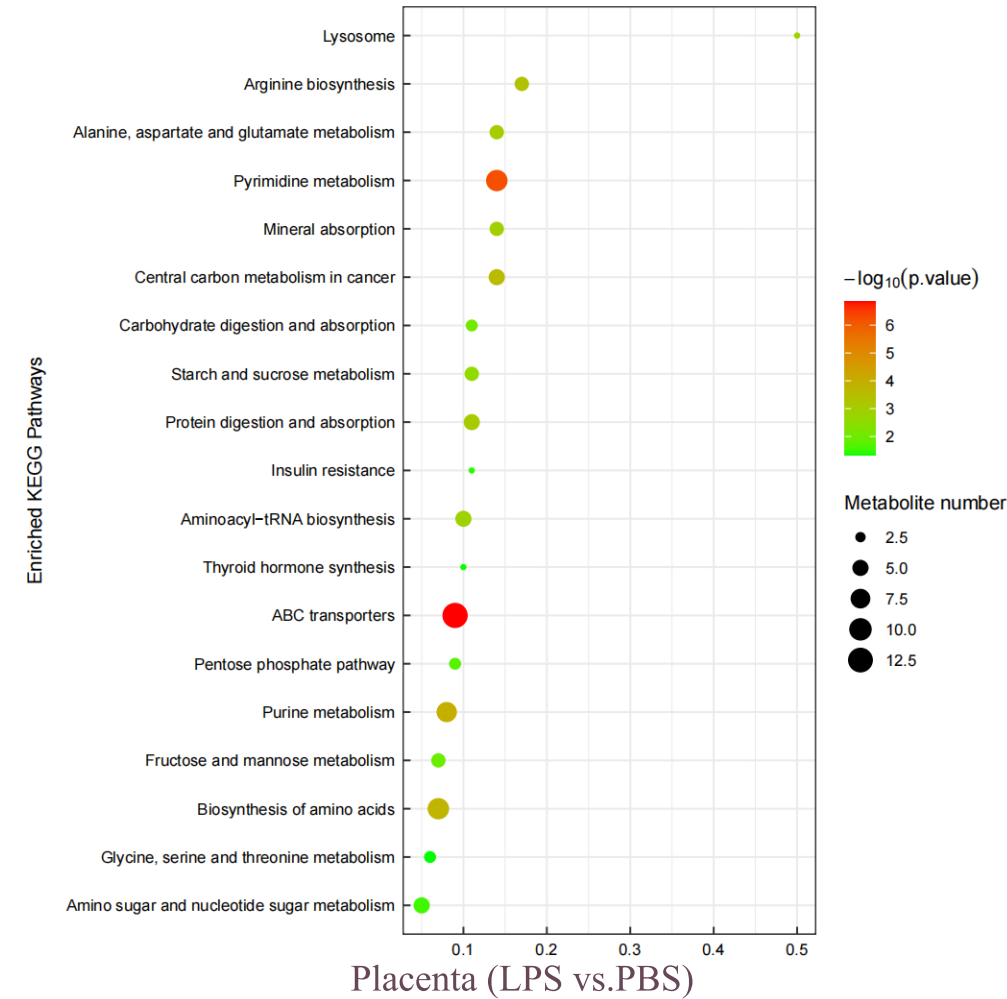

B

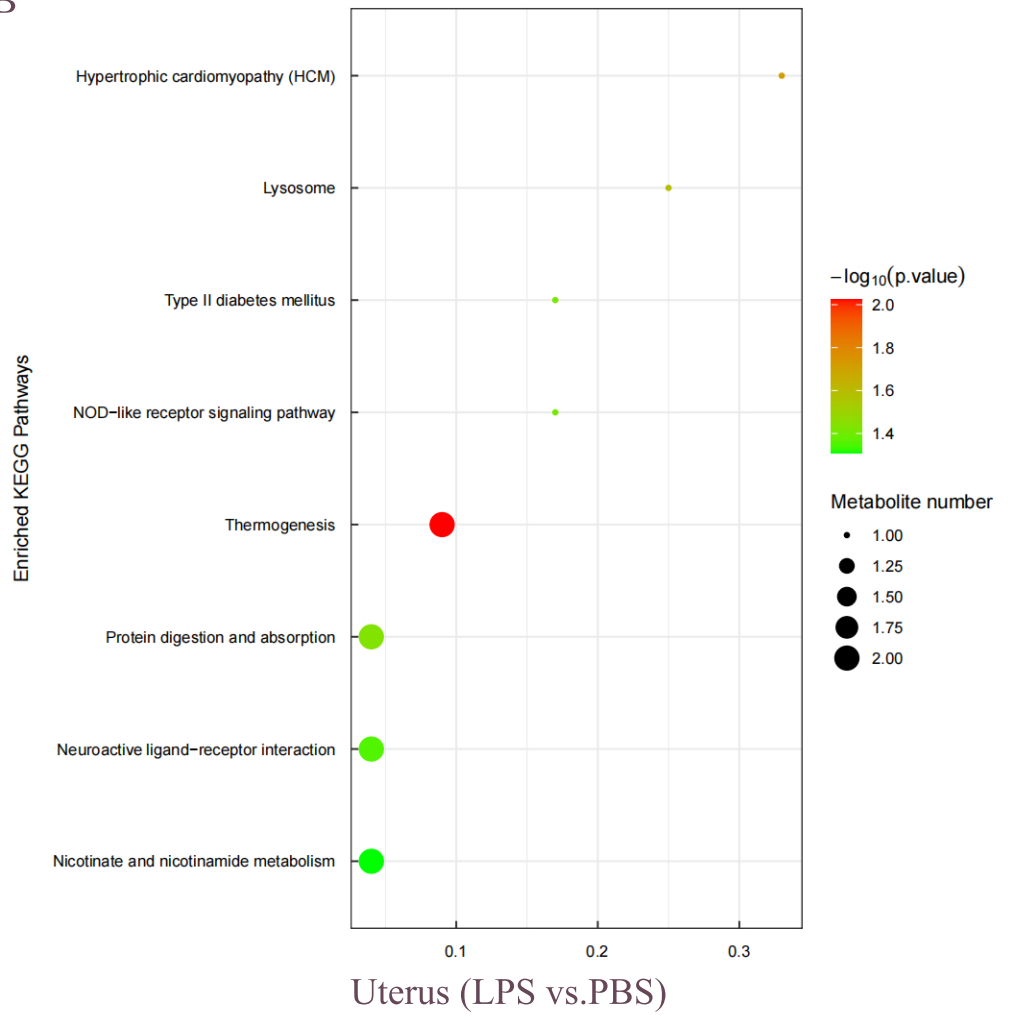

Supplement: Supplementary Figure 12 — Network diagram of DEMs in positive and negative ion mode. (A) Placenta (POS-LPS vs. PBS). (B) Placenta (NEG-LPS vs. PBS). (C) Uterus (POS-LPS vs. PBS). (D) Uterus (NEG-LPS vs. PBS). Top 30 hub DEMs (P adj < 0.05, |log2(fold change)| > 1) identified using CytoHubba. Each circle in the figure represents a metabolite, and the lines between the circles indicate interactions between metabolites. The color of a circle reflects the number of interactions that the corresponding metabolite has, with a darker color indicating a higher degree of connectivity. [file DataSheet_10.pdf]

A

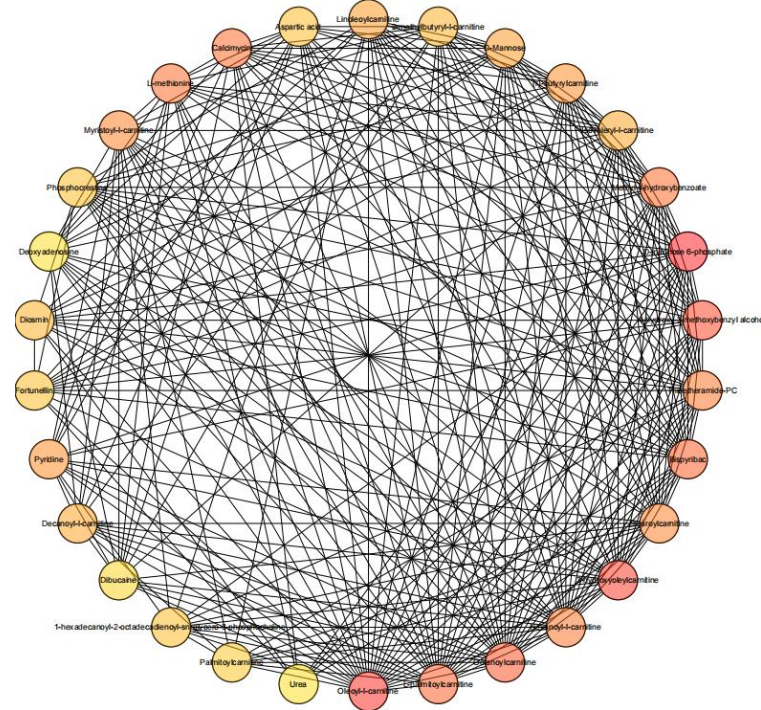

Uterus (POS)

Placenta (POS)

B

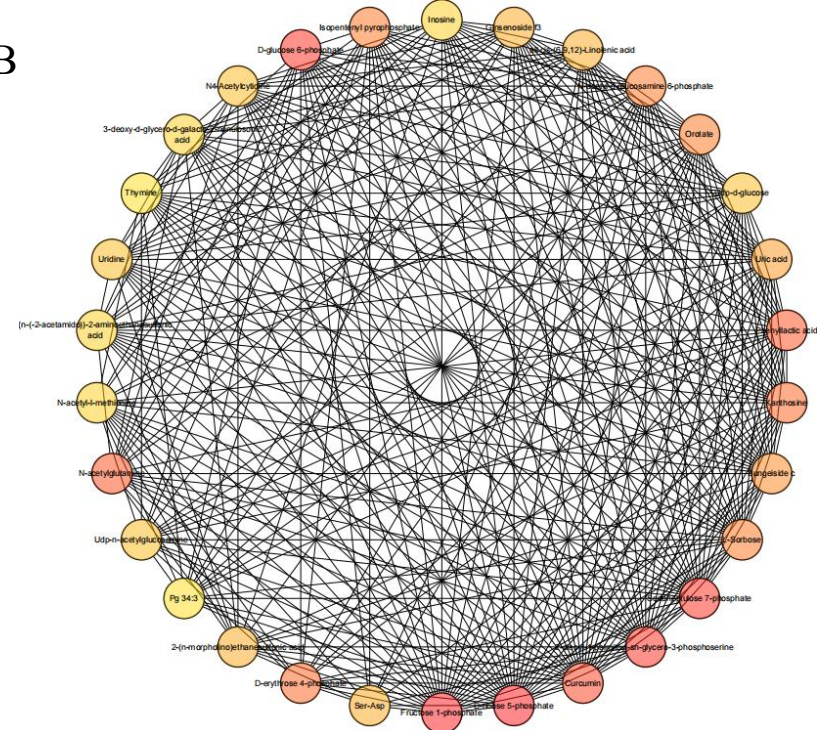

Uterus (NEG)

Placenta (NEG)

C

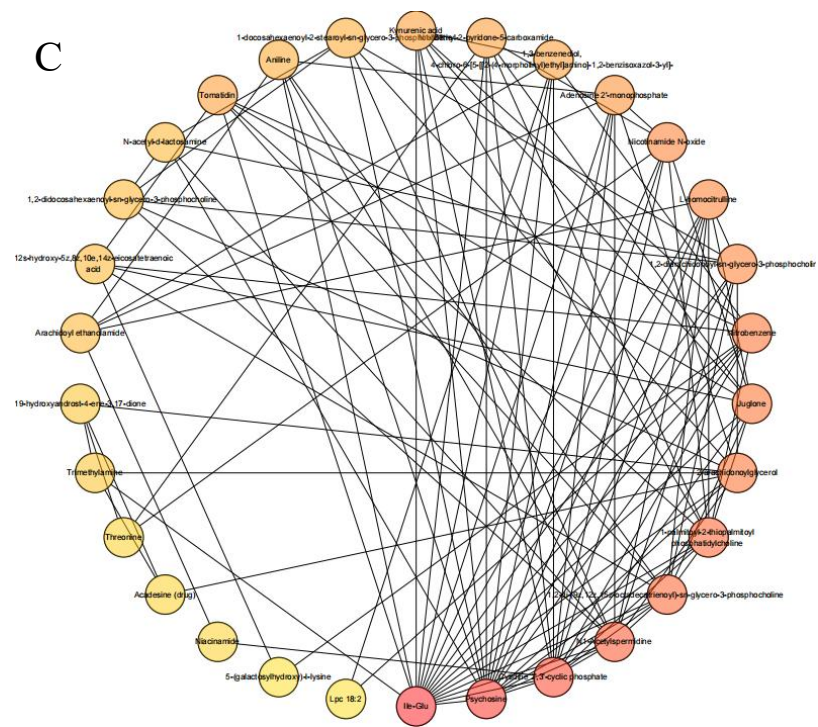

D

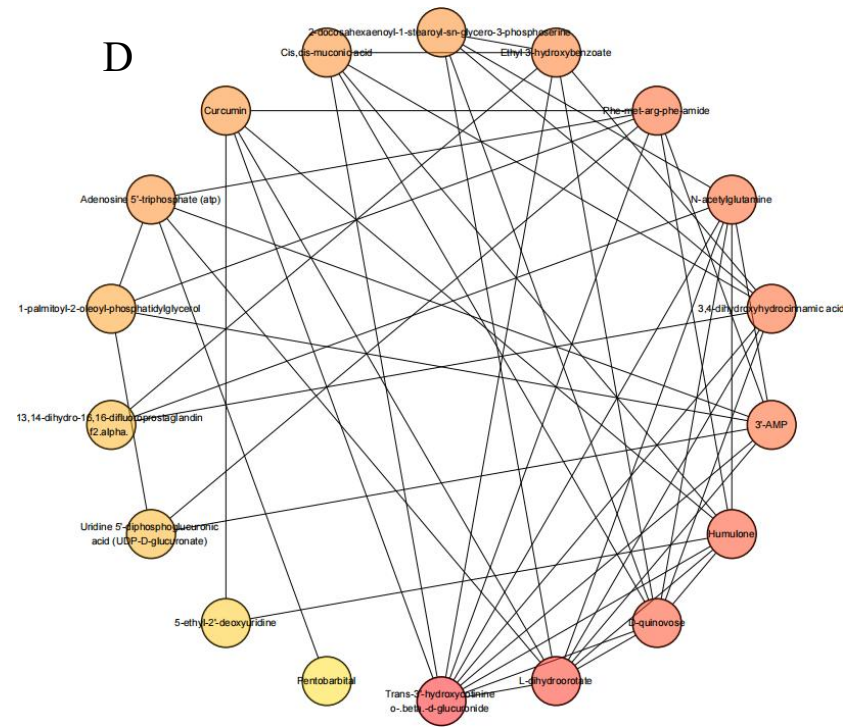

Supplement: Supplementary Figure 13 — Hierarchical clustering heatmap of Spearman correlation analysis of DEGs AND DEMs. (A) Placenta (LPS vs. PBS). (B) Uterus (LPS vs. PBS). In the hierarchical clustering heat map, each row represents a DEM, and each column represents a DEG. The left branch represents the difference in pairs. The upper branch represents the result of cluster analysis of DEGs. Each lattice in the hierarchical clustering heatmap contains two kinds of information (correlation coefficient r and P-value). The correlation coefficient r is represented by color, r> 0 represents a positive correlation, and r < 0 represents a negative correlation. A deeper color indicates a stronger correlation. [file DataSheet_11.pdf]

A

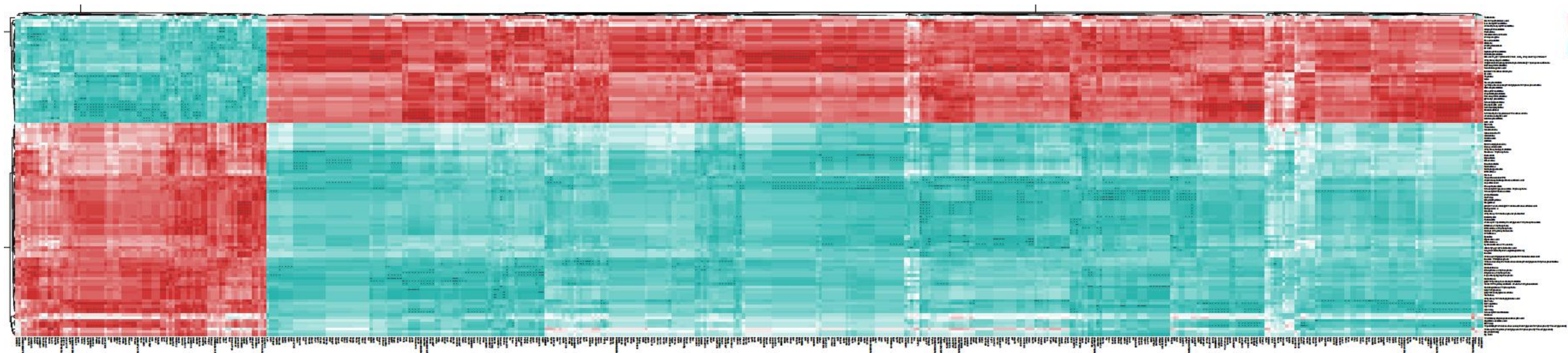

B

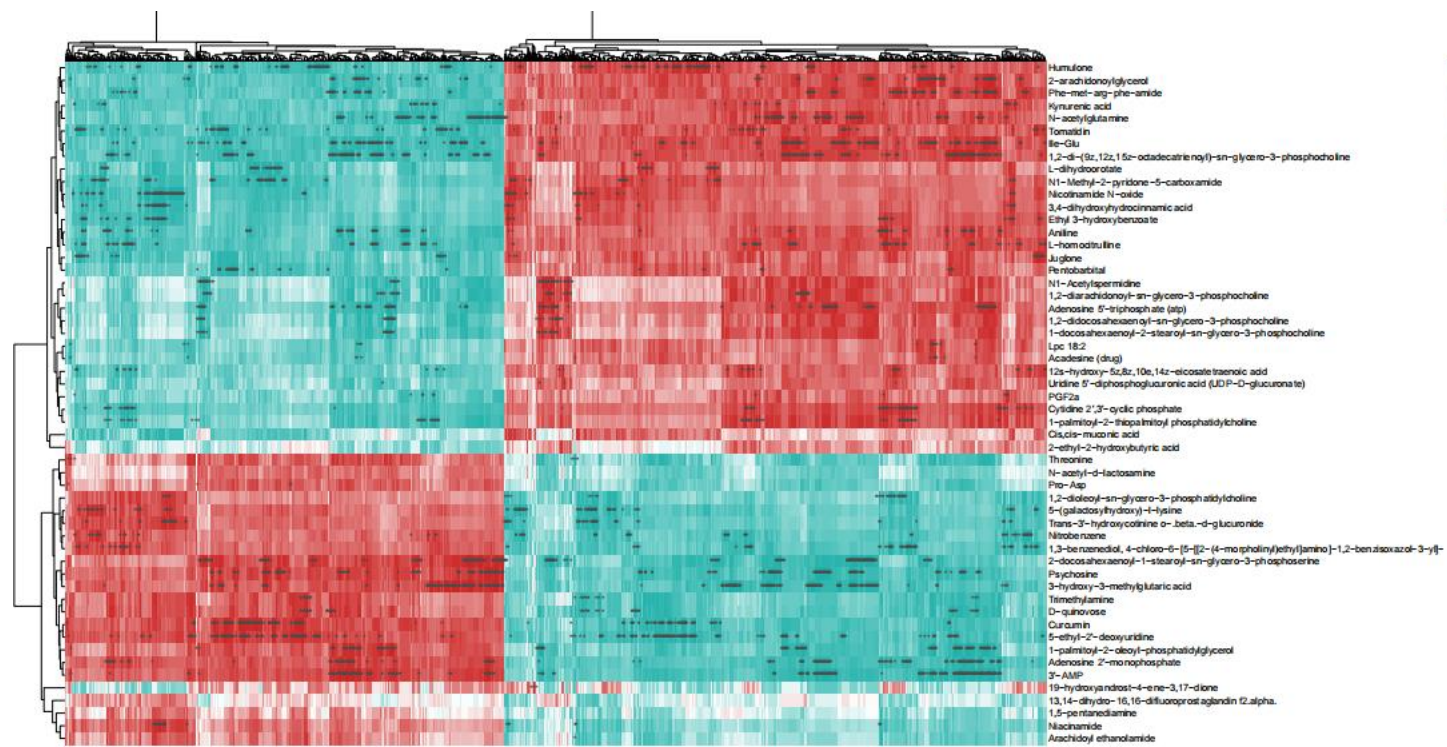

Supplement: Supplementary Figure 14 — Histogram of common KEGG enrichment pathway of DEGs and DEMs. (A) Placenta (LPS vs. PBS). (B) Uterus (LPS vs. PBS). Each column in the figure represents a KEGG pathway, with different colors representing different omics, among which blue represents transcriptome and orange represents metabolomics. The ordinate represents the name of the pathway, and the abscissa represents the significance of pathway enrichment. Columns with greater height indicate a higher level of enrichment of biological pathways in the measured samples and a more obvious change in activity. [file DataSheet_12.pdf]

A

KEGG Pathways

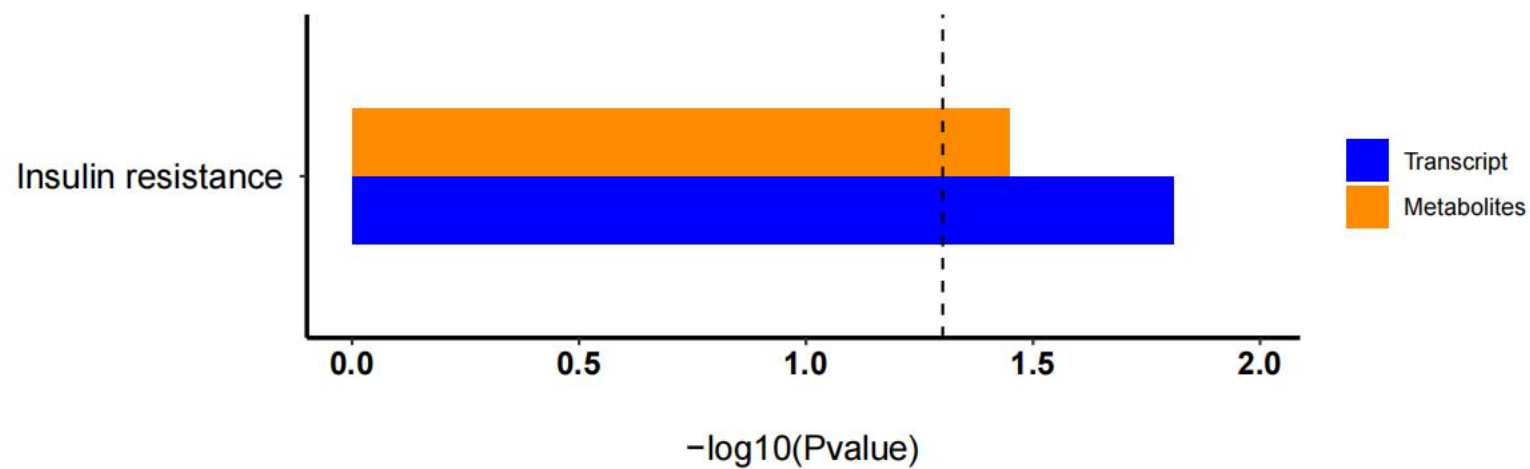

B

KEGG Pathways

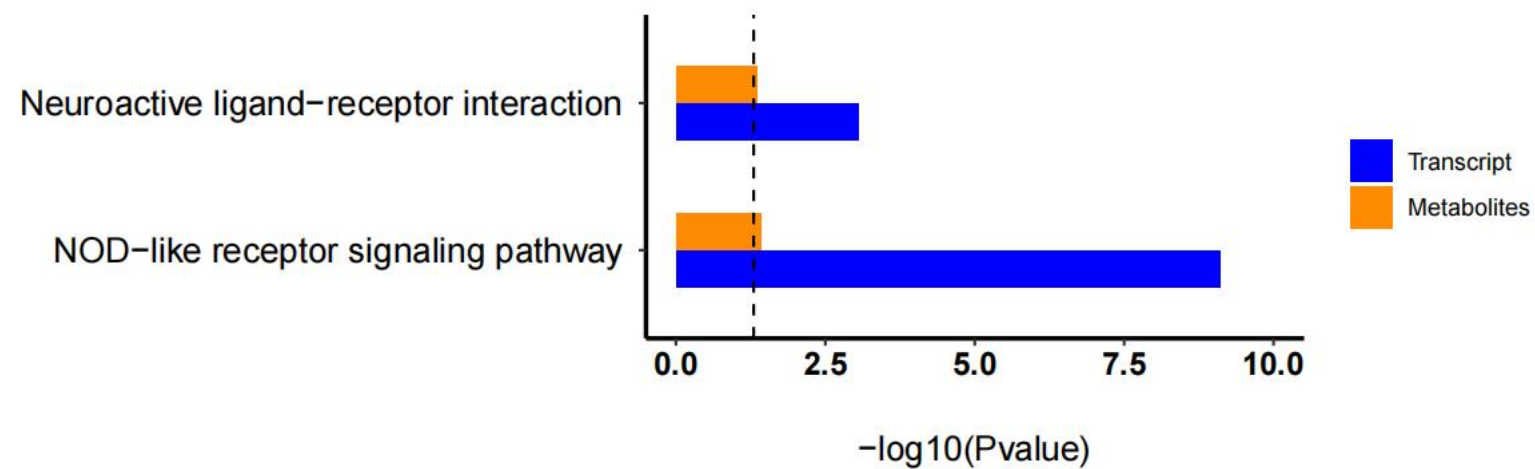

Supplement: Supplementary Figure 15 — KEGG pathway annotation map of DEGs and DEMs. (A, D, E) Uterus (LPS vs. PBS). (B, C) Placenta (LPS vs. PBS). The box in the figure represents the gene product, and the circle represents the metabolite. The red or green background represents DEGs or DEMs. [file DataSheet_13.pdf]

A

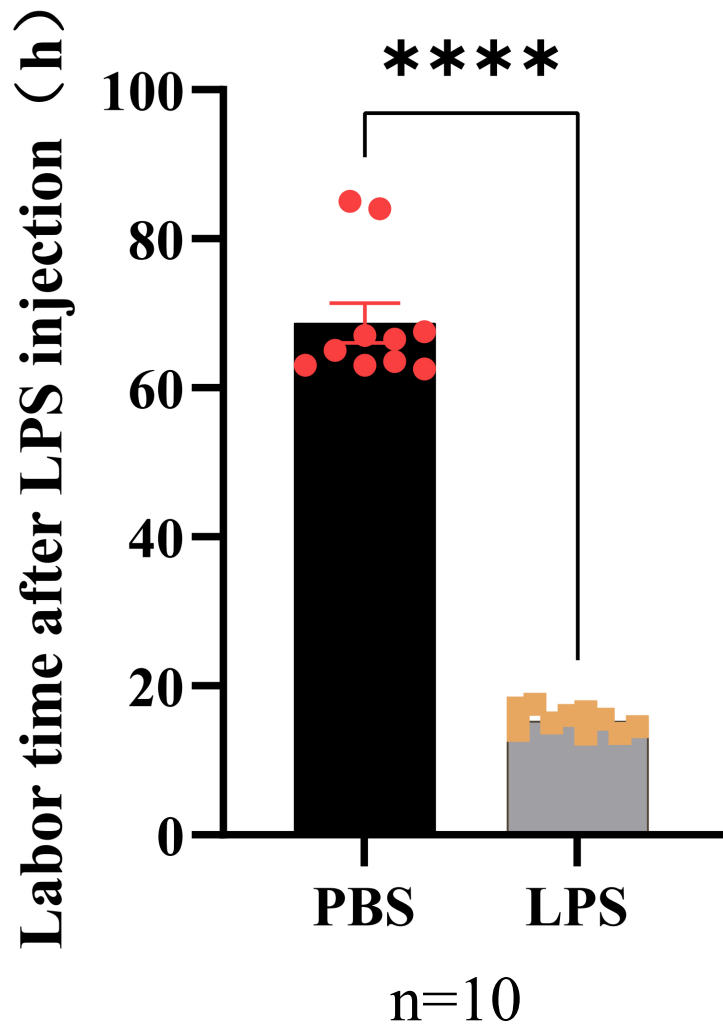

B

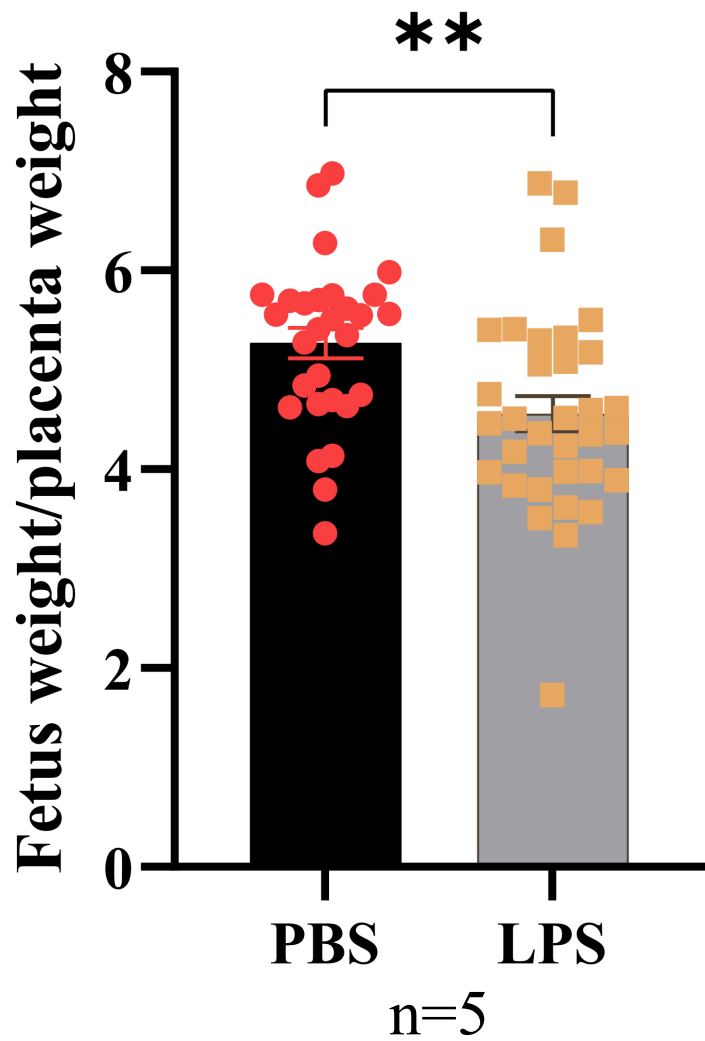

Supplement: Supplementary file 16 [file DataSheet_15.pdf]
