# Supplementary material for: Integration of transcriptomics and metabolomics reveals the responses of the maternal circulation and maternal-fetal interface to LPS-induced preterm birth in mice
Source: Front Immunol. 2023 Aug 15;14:1213902. doi: 10.3389/fimmu.2023.1213902 (PMC10464907; doi:10.3389/fimmu.2023.1213902)
Supplement: Supplementary Figure 16 — Correlation analysis network diagram of representative DEGs and DEMs. (A) Placenta (LPS vs. PBS). (B) Uterus (LPS vs. PBS). In the figure, red circles represent DEGs or DEMs (P adj < 0.05, |log2(fold change)| > 1), and the lines represent the correlation between the two. [file DataSheet_14.pdf]

A

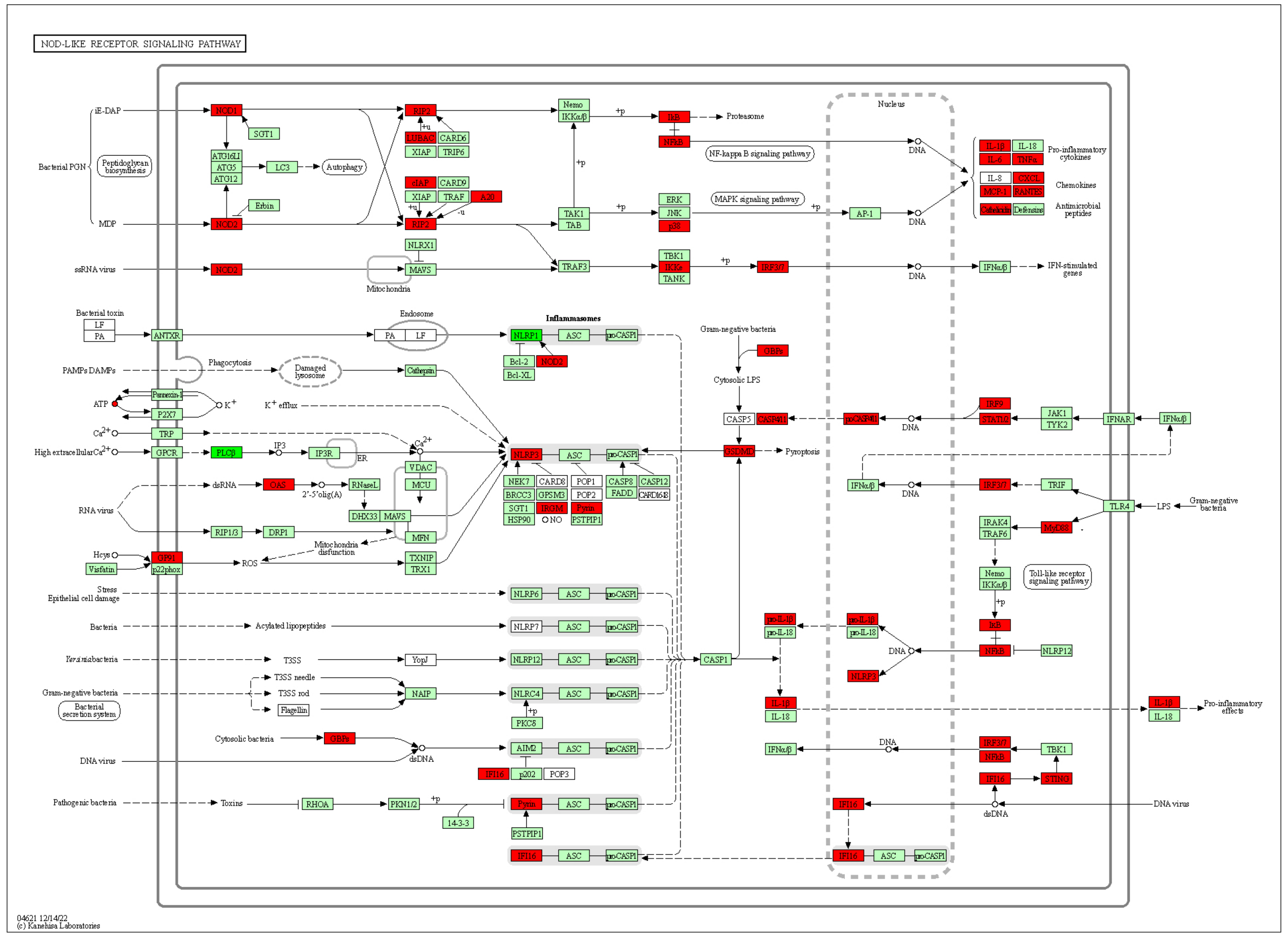
NOD-LIKE RECEPTOR SIGNALING PATHWAY

B

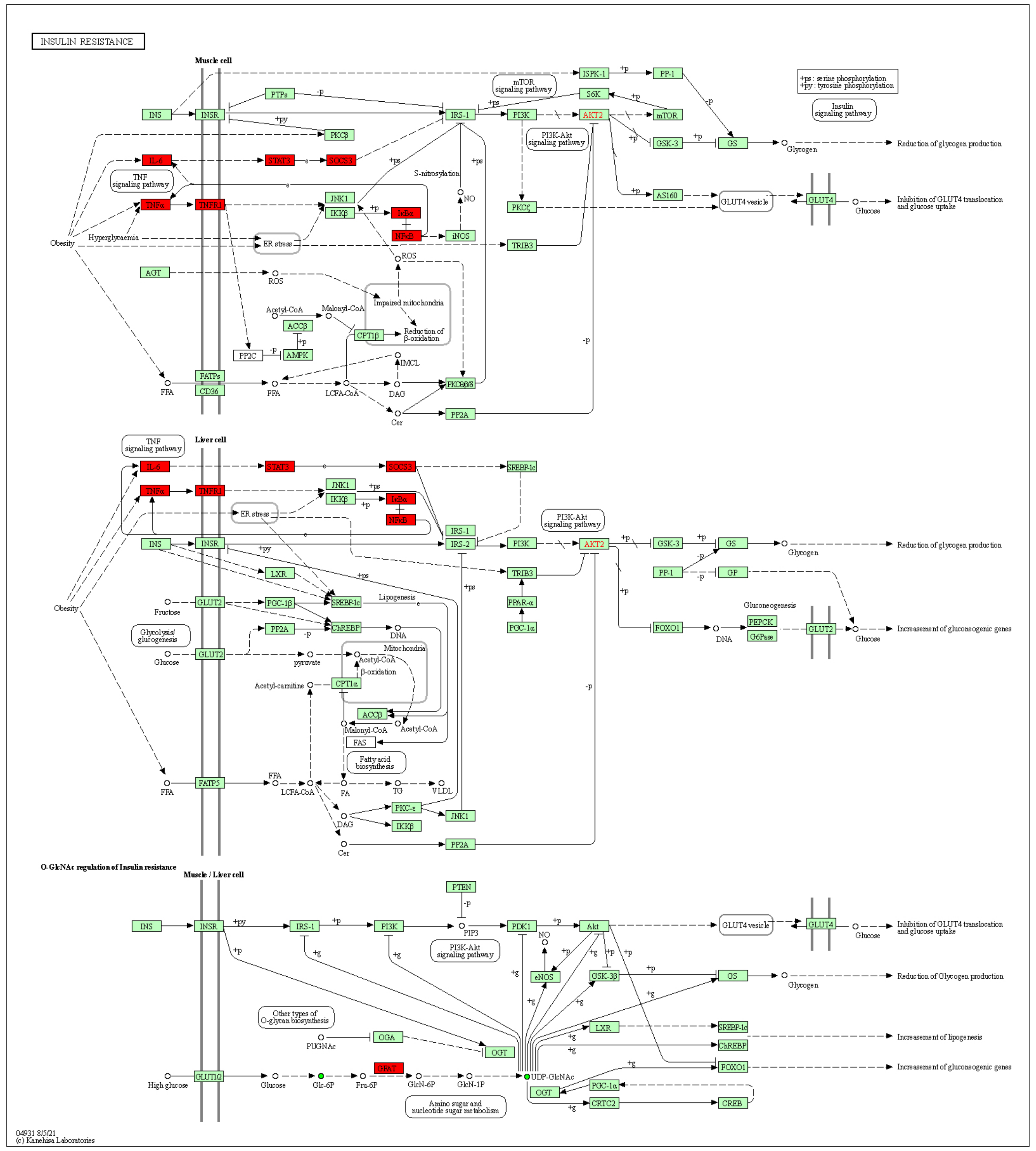
INSULIN RESISTANCE

C

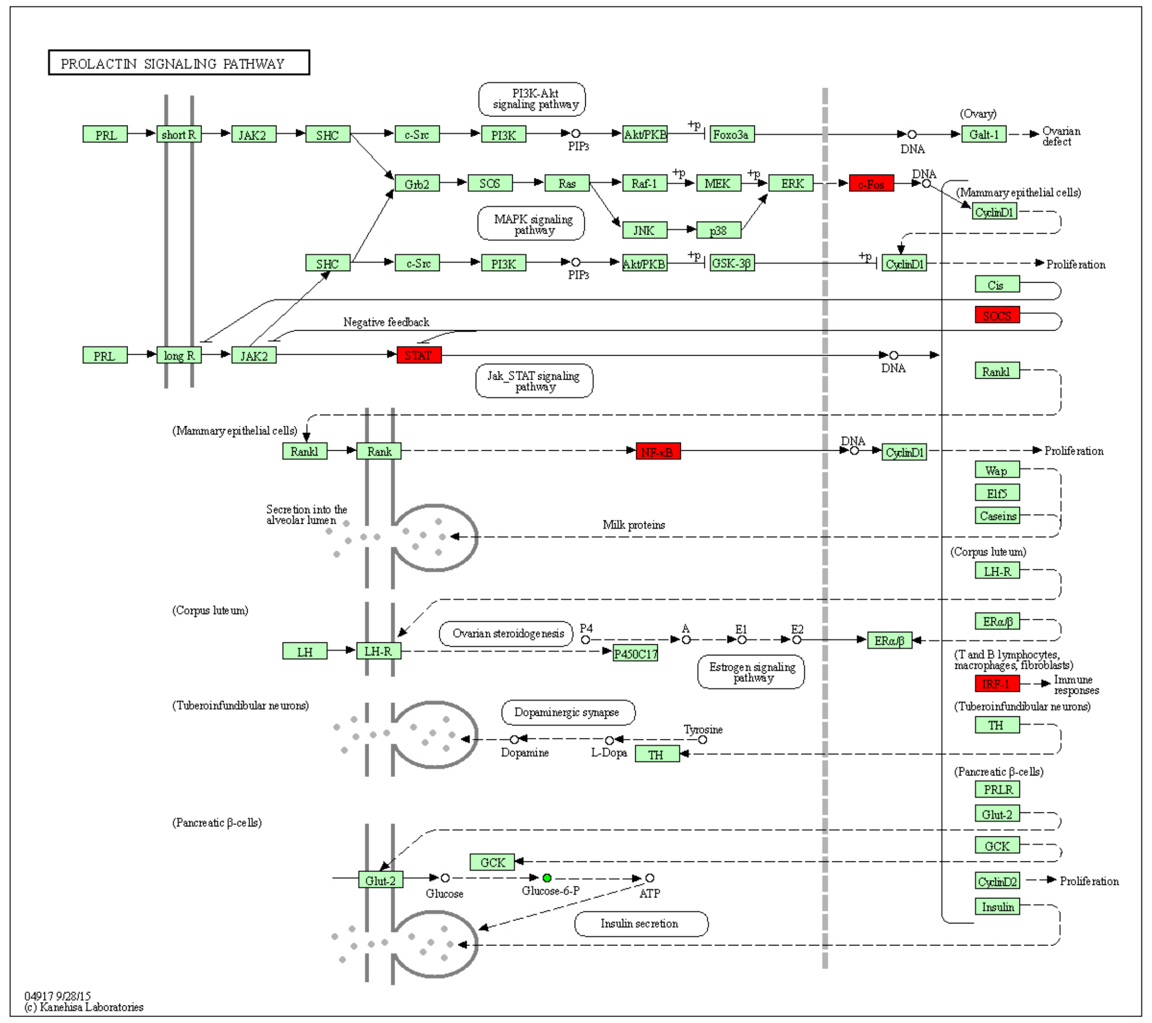
PROLACTIN SIGNALING PATHWAY

D

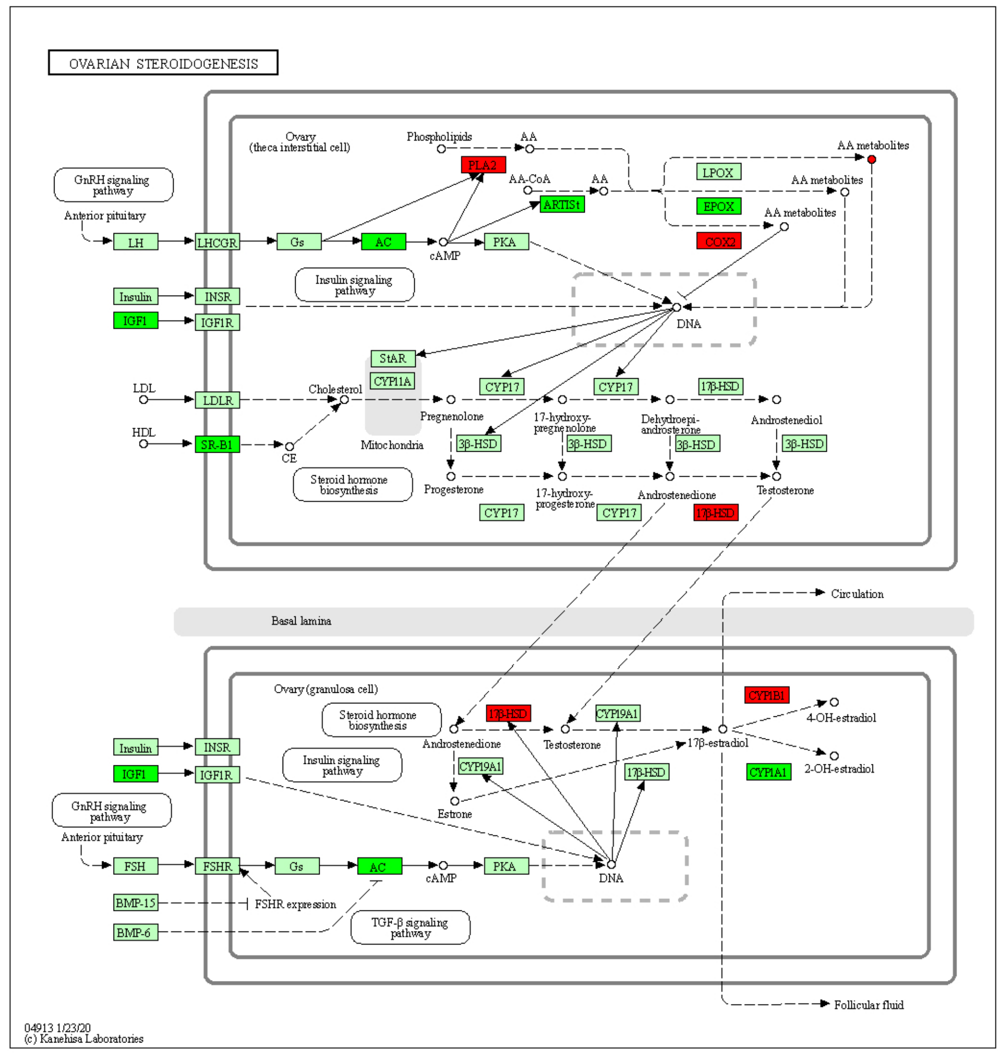
OVARIAN STEROIDOGENESIS

E

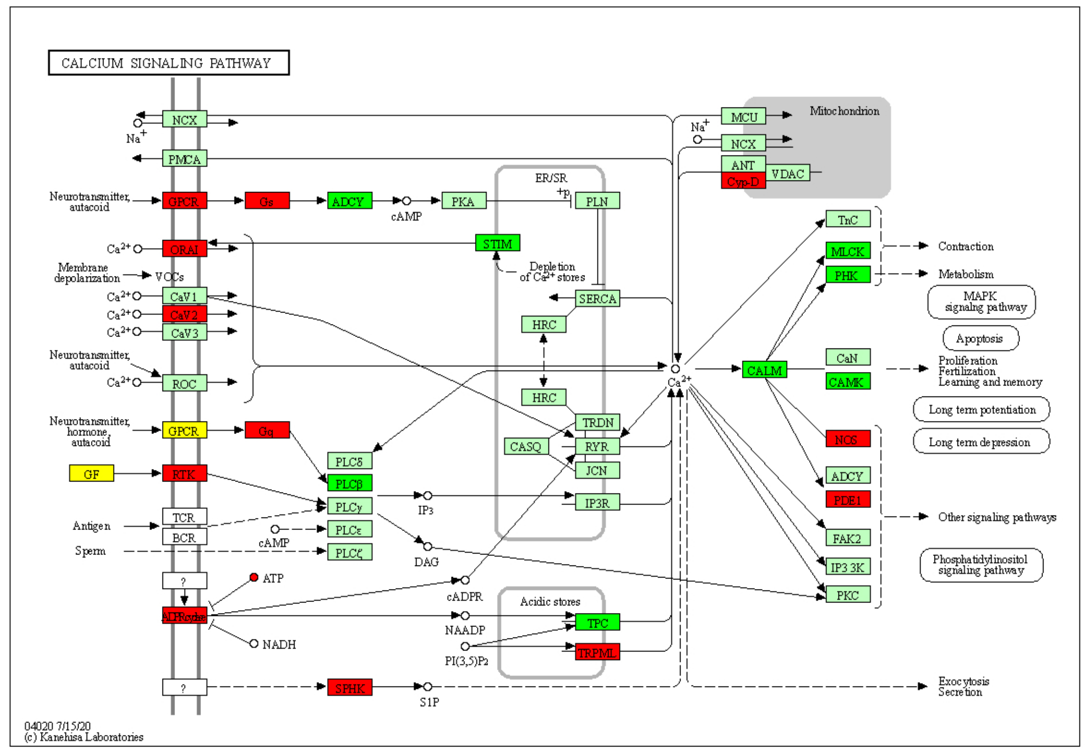
CALCIUM SIGNALING PATHWAY
